# Archetypal Architecture Construction, Patterning, and Scaling Invariance in a 3D Embryoid Body Differentiation Model

**DOI:** 10.3389/fcell.2022.852071

**Published:** 2022-04-27

**Authors:** Olga Gordeeva, Andrey Gordeev, Pavel Erokhov

**Affiliations:** ^1^ Koltzov Institute of Developmental Biology, Russian Academy of Sciences, Moscow, Russia; ^2^ National Institutes of Health’s National Library of Medicine, Bethesda, MD, United States

**Keywords:** embryoid bodies (EBs), pluripotent stem cell, differentiation, morphogenesis, patterning, self-organisation

## Abstract

Self-organized patterning and architecture construction studying is a priority goal for fundamental developmental and stem cell biology. To study the spatiotemporal patterning of pluripotent stem cells of different origins, we developed a three-dimensional embryoid body (EB) differentiation model quantifying volumetric parameters and investigated how the EB architecture formation, patterning, and scaling depend on the proliferation, cavitation, and differentiation dynamics, external environmental factors, and cell numbers. We identified three similar spatiotemporal patterns in the EB architectures, regardless of cell origin, which constitute the EB archetype and mimick the pre-gastrulation embryonic patterns. We found that the EB patterning depends strongly on cellular positional information, culture media factor/morphogen content, and free diffusion from the external environment and between EB cell layers. However, the EB archetype formation is independent of the EB size and initial cell numbers forming EBs; therefore, it is capable of scaling invariance and patterning regulation. Our findings indicate that the underlying principles of reaction-diffusion and positional information concepts can serve as the basis for EB architecture construction, patterning, and scaling. Thus, the 3D EB differentiation model represents a highly reproducible and reliable platform for experimental and theoretical research on developmental and stem cell biology issues.

## Introduction

Pluripotent stem cells develop into all tissues and organs after their reintegration with an early embryo or *in vitro* recapitulate the developmental events after their self-assembly and self-organization in 3D cellular structures (Doetschman et al., 1985; Robertson et al., 1986; Morris et al., 2012). The 3D models based on pluripotent stem cells–embryoid bodies (EBs), blastoids, gastruloids, bi- (pluripotent and trophoblast) and tri-compartmental (pluripotent, trophoblast and extraembryonic endoderm) embryo-like aggregates (ET- and ETX-embryos, respectively)—facilitate studying the spatiotemporal patterning and dynamics of cellular processes under various experimental conditions (Bratt-Leal et al., 2009; Giobbe et al., 2012; Van Den Brink et al., 2014; Harrison et al., 2017; Flamier, 2018; Rivron et al., 2018; Rossi et al., 2018; Shahbazi et al., 2019; Sozen et al., 2019; Kim et al., 2020; Kagawa et al., 2021; Yu et al., 2021). These 3D cellular models can reproduce the complex architecture of mammalian embryonic structures and are considered promising *in vitro* models for fundamental studies, drug discovery, and toxicological screening. The combination of 3D embryonic cell models and new experimental technologies allows more profound exploration of fundamental questions in mammalian and human developmental biology, such as self-organization, symmetry breaking, patterning, and scaling, which are also relevant to teratology and developmental toxicology (Bauwens et al., 2008; Van Den Brink et al., 2014; Deglincerti et al., 2016; Warkus et al., 2016; Dutta et al., 2017; Kang et al., 2017; Tewary et al., 2017; Sozen et al., 2021; van den Brink and van Oudenaarden, 2021; Wahlin et al., 2021).

Previous studies facilitated the development of efficient protocols for *in vitro* pluripotent stem cell differentiation to reconstruct the developmental trajectories for multiple cell types (Oh and Jang, 2019; El Azhar and Sonnen, 2021; van den Brink and van Oudenaarden, 2021). Numerous 2D and 3D engineered embryonic cell models allowed the investigation of the developmental mechanisms at different levels previously studied only in animal embryos (Camacho-Aguilar and Warmflash, 2020). However, regardless of the significant progress in creating and studying embryo-like models, there are remain insufficiently explored possible 3D morphological archetypes generated by combining different embryonic cells and the cellular mechanisms coordinating the architecture construction (Renner et al., 2017; Sozen et al., 2018; Zheng et al., 2019; van den Brink et al., 2020; Zhu and Zernicka-Goetz, 2020; El Azhar and Sonnen, 2021; Guo et al., 2021; Jerber et al., 2021; Molè et al., 2021). Therefore, further studies of the 3D embryonic cell models with different “starting” cell populations, including cancer cells, are needed for disclosing the mechanisms of morphogenesis and architecture construction in different environments. Moreover, addressing these issues is critical for standardizing protocols that reduce variability and improve reproducibility in the embryoid and organoid models for basic and pharmacological research.

In the presented study, the standardized and well-scalable 3D EB differentiation model was used to analyze the architecture creation *via* self-organization, morphogenesis, patterning, and differentiation of the embryonic stem (ESC), embryonic germ (EGC), and teratocarcinoma (ECC) cells, which differ in genetic and epigenetic states and spontaneous differentiation potentials *in vitro* and *in vivo* (Rizzino and Crowley, 1980; Andrews, 1988; Wobus and Boheler, 2005; Sharova et al., 2007; Shim et al., 2008; Weinberger et al., 2016; Kelly and Gatie, 2017; Liu et al., 2020). We used these cell lines to test the hypothesis about the equifinality in the EB architecture construction, i.e., whether these cells of different origins are capable of forming similar morphological structures mimicking early developmental events. We sought to identify the basic principles and specific features in creating the architecture of EBs, formed by cell lines of various origins, analyzing proliferation, differentiation, and cavitation dynamics in different environments. Pursuing our goals, we identified three similar spatiotemporal patterns in the EB architecture regardless of cell origin, which constitute the EB archetype and mimick the embryonic patterns at the pre-gastrulation stages. We found that the EB patterning strongly depends on external environmental factors and the position of cell layers, but not on the initial number of cells forming EBs, and these general properties contribute to scaling invariance and patterning regulation. We assume that the basic principles of both reaction-diffusion (RD) (Turing, 1952) and positional information (PI) (Wolpert, 1981) concepts can serve as the basis for the EB architecture patterning. Therefore, the 3D EB differentiation model can be used as a highly effective and reliable platform for experimental, theoretical, and computational analyses of cell and developmental biology issues.

## Materials and Methods

### Cell Lines and Culture

Mouse R1 embryonic stem cells (ESCs) and EGC-10 embryonic germ cells (EGCs) were kindly provided by Dr. A. Nagy (Mount Sinai Hospital, Toronto, Canada) and Dr. A. McLaren (WTCR Institute of Cancer and Developmental Biology, Cambridge, UK). The F9 embryonic teratocarcinoma cell line (ECCs) was obtained from the Russian Cell Culture Collection (http://www.rccc.cytspb.rssi.ru/). The ESCs, EGCs, and ECCs were routinely cultivated on gelatin-coated dishes (3%, Sigma-Aldrich) in fetal bovine serum-containing media (FBS; (SH30071.04, HyClone) at 5% CO_2_ and 37°C, as described previously (Gordeeva et al., 2019). For undifferentiated ESCs and EGCs, media was supplemented with leukemia inhibitory factor (LIF, L-5158; 1000 U/ml, Sigma-Aldrich). All cell lines were routinely tested using MycoFluor™ *Mycoplasma* Detection Kit (M7006; Invitrogen). Cells were routinely passaged as single cells every 3 days using 0.05% Trypsin-EDTA (25-300-120; Gibco). To analyze the numbers of viable cells, the ESCs were stained with 0.4% trypan blue dye (15250-061; Gibco/Thermo Fisher Scientific) and non-stained cell numbers were counted using an Improved Neubauer Hemocytometr (Hausser Scientific, Horsham, PA, United States).

### Mouse Embryo Experiments

All animal experiments were approved by the Ethics Committee of Institute of Developmental Biology of Russian Academy of Sciences and performed in accordance with the Russian Federation legislation (Order of the Ministry of Health and Social Development of the Russian Federation No 708n, 28 August 2010) based on the European Convention for the Protection of Vertebrate Animals Used for Experimental and Other Scientific Purposes. C57Bl/6 mice at the age of 10–12 weeks were obtained from the Animal Breeding Facility-Branch “Pushchino” of the Institute of Bioorganic Chemistry, Russian Academy of Sciences. To obtain the embryos, females were mated with males overnight and vaginal plugs were tested on the following morning (embryonic stage E0.5). Fertilized females were sacrificed by cervical dislocation and embryos at the morula (E2.5) and blastocyst (E3.5 and E4.5) and egg cylinder (E6.5) stages were recovered from the oviduct and uterus and placed in the M2 medium with HEPES (M7167; Sigma-Aldrich). The epiblasts with adjacent extraembryonic endoderm of E6.0–6.5 embryos were isolated after dissection of the uterus tissues, trophectoderm and ectoplacental cone.

### EB Generation and Differentiation

The EBs were generated using the “hanging drop” method by placing the cell suspension drops (usually 500 cells per drop, and 50, 100, 500, and 1,000 cells for differently sized EBs) on the dish lids (10-cm dish, Greiner Bio-One International GmbH) for 48 h. After formation, the ESBs, EGBs, and ECBs, formed by ESCs, EGCs, and ECCs, respectively, were collected and cultured in low adhesion plates (Greiner Bio-One International GmbH and Nunclon Sphera/Thermofisher Scientific) in LIF-free media in the following 10 days. To maintain the EB spherical shape, the low adhesion plates with EBs were continuously shacked during the cultivation.

To study the growth and differentiation dynamics, the EBs were cultured in FBS- and KSR-media for 10 days. FBS-media consisted of the DMEM (SH30285.01) supplemented with 2 mM L-glutamine (SH30034.01), 1% non-essential amino acids (SH3023801), 15% Characterized Fetal Bovine Serum (SH30071.04) (all from HyClone), and 0.1 mM β-mercaptoethanol (M3148; Sigma-Aldrich). KSR-media consisted of the KnockOut DMEM (10829018), 2 mM L-glutamine (A2916801), 1% non-essential amino acids (11140050), 15% KnockOut Serum Replacement (KSR; A3181501) (all from Gibco/ThermoFisher Scientific), and 0.1 mM β-mercaptoethanol (Sigma-Aldrich). LIF (1000 U/ml, Sigma-Aldrich) and all-trans-retinoic acid (10^−6^ M RA; R2625, Sigma-Aldrich) were added to study their influence on the EB growth and differentiation for 5 days.

To study the patterns’ regulation, merging EBs at different stages were placed in hanging drops on the 60 mm dish lid (Nuclon) or in culture medium drops covered with mineral oil (Sigma-Aldrich) in 35 mm dishes (Nuclon) for 1–2 days (5 EBs per stages for each cell line; three experiments; N = 60 for each cell line). EBs separated by a scalpel under a stereomicroscope (5 EBs per stages for each cell line; three experiments; N = 45 for each cell line) were cultured in 4-well plates (Nuclon) for 1–2 days and then analyzed. To study pattern desorganization-reconstraction, individual EBs at day10 (5 EBs/per stage for each cell line; three experiments; N = 60 for each cell line) were dissociated into single cells using 0.05% Trypsin-EDTA (25-300-120; Gibco) at 37°C for 5 min, followed by gentle mechanical dissociation with a pipette. For *de novo* EB formation, the cell suspension from individual EBs was placed in hanging drops (100 cells/per drop) on the lid of 60 mm dish (Nuclon) for 72 h.

### EB Growth and Patterning Analysis

To evaluate EB growth dynamics and patterning, the EBs (24 EBs/per stage; three experiments; N = 288 for each cell line) cultivated in 12 or 96-well plates with a low-attachment surface (Greiner Bio-One International GmbH and Nunclon Sphera; Nuclon) and imaged at the studied stages. The EB images were captured using Olympus CK40 inverted microscope equipped with a Camedia C-4040 camera (Olympus). EB diameters were measured in 2-8 directions for average calculation using ImageJ/Fiji software (https://imagej.nih.gov/ij/) with prior set up absolute scale calibration (see Supplementary Methods). EB volumes, volumetric pattern sizes and their ratios were calculated using Excel.

### Histological and Electron Microscopy Analyses

To prepare semi-thin and ultrathin sections, EBs were fixed by 2.5% glutaraldehyde (Sigma-Aldrich) in 100 mM sodium cacodylate buffer (Serva, Germany), pH 7.0 for 24 h at 4°C and then post-fixed with 1% osmium tetroxide (Serva, Germany) in 100 mM cacodylate buffer, pH 7.0 (all from Sigma-Aldrich) within 1 h at 4°C. After dehydration procedures in serial ethanol and acetone solutions and impregnation in resin, the specimens were embedded into the Spurr’s resin (Spurr kit, Sigma) or Araldite (Fluka). The EB sections were prepared using Ultrotome 3 (LKB, Germany). Semi-thin sections were stained with toluidine blue in 1% sodium tetraborate (Sigma-Aldrich). Ultra-thin sections were placed on the grids and stained with aqueous uranyl acetate (Merck, Germany) and lead citrate (Serva, Germany). The sections were examined under a JEM 100CXII electron microscope (Jeol, Japan).

### EdU-Labeling and Caspase Activity Assays

For the analysis of cells in the S-phase of the cell cycle, the EBs were incubated with 10 μM 5-ethynyl-2-deoxyuridine (EdU) for 1 h at 37°C and 5% CO_2_ and then with a reaction solution from the Click-iT EdU Imaging Kit with Azide-Alexa 488 (C10083; Molecular Probes) according to the protocol recommended by the manufacturer. After the completion of the Click-iT EdU reaction, the EBs were fixed with 3% paraformaldehyde (P6148; Sigma-Aldrich) in PBS, washed with PBS and stained with Hoechst 33342 (1 μg/ml; H3570; Molecular Probes). EBs were cleared in glycerol-PBS, placed on 18-well µ-Slides (81826; Ibidi, Martinsried, Germany) in a mounting medium (50001; Ibidi) and imaged under a Leica TSC SPE confocal microscope.

The Vybrant FAM Poly Caspases Assay Kit (V35117, Molecular Probes) for the detection of caspase-1, -3-9 activity was used according to the manufacturer’s recommendations. The EBs were incubated in culture media containing FLICA reagent (FAM-VAD-FMK poly caspases reagent) for 1 h at 37°C and 5% CO_2_ and Hoechst 33342 (1 μg/ml) and propidium iodide (PI; 1 μg/ml) were added to the culture media for 15 min. After washing with PBS, the EBs were mounted on 15-well µ-Slides (81506; Ibidi) and immediately examined under a confocal microscope.

### Alkaline Phosphatase Activity and Immunofluorescence Analyses

For alkaline phosphatase activity (ALP) assay, EBs were fixed by 2% paraformaldehyde in PBS, pH 7.0 within 15 min, washed with PBS and incubated in a solution containing 10 ml 0.02 M Tris-HCl buffer, pH 8.7 (T1503), 1 mg Naphthol-AS-B1-phosphate (N2125), and 5 mg Fast Red TR dye (305464) (all reagents from Sigma-Aldrich) at 37°C for 1 h.

For immunofluorescence analysis, mouse embryos and EBs were fixed with 3% paraformaldehyde in PBS for 1 h, washed with PBS, and then permeabilized and blocked with 0.5% Triton X-100 (T9284; Sigma-Aldrich) and 3% Bovine Serum Albumin, Fraction V (85040C; Sigma-Aldrich) in PBS within 1–3 h. Primary antibodies were incubated in PBS with 0.25% Tween 20 (P1379; Sigma-Aldrich) at 4°C overnight. The following dilutions of primary antibodies were used: Oct4 (1:150; rabbit IgG, sc-9081, Santa Cruz Biotechnology), Gata4 (1:150; goat IgG, sc-1237, Santa Cruz Biotechnology), and ZO-1 protein (1:100, rabbit IgG, 61–7,300, Invitrogen). After extensive washing in PBS, secondary Alexa Fluor 488 and 594 chicken anti-rabbit antibodies (1:800; A-21441 and A-21442, Molecular Probes), Alexa Fluor 488 donkey anti-goat antibodies (1:800; A-11055, Molecular Probes) and Alexa Fluor 594 chicken anti-goat antibodies (1:800; A-11058, Molecular Probes) were applied for 3–4 h. After staining and extensive washing, the embryos and EBs were cleared in increasing concentrations of glycerol in PBS (30, 50, 75, and 90%), mounted on 18-well µ-Slides (Ibidi) in a glycerol-based mounting medium (Ibidi), and imaged.

### Confocal Microscopy Image Acquisitions, Processing, and Quantitative Analysis

The embryos and EBs were placed on the 18- and 15-well µ-Slides (Ibidi) and studied under Leica TSC SPE and SP5 confocal microscopes using the 405, 488, and 596 lasers and HCX PL APO CS 20.0 × 0.70 IMM UV and 40.0 × 1.25 OIL UV objectives. Z-stacks of optical sections were acquired with 0.3–1 µm z-steps at 100–120 µm depth of specimen scanning. To generate the 3D volume reconstruction for EBs and embryos, the confocal z-stacks were processed using 3D Viewer Plugins of ImageJ/Fiji software.

To analyze the proliferative cell domain, the numbers of EdU-labeled and the total Hoechst-stained nuclei in the EBs were counted using the 2D Automated Cell Counter tool in ImageJ software (https://imagej.nih.gov/ij/plugins/cell-counter.html) for confocal stacks, as previously described (Gordeeva and Gordeev, 2021). The cell counts were based on the object-level identification of Hoechst- and EdU-stained nuclei in each plane of confocal z-stack, which were counted in two separate channels. For the segmentation of merged nuclei, each image was run through the watershed algorithm and manually corrected. The square range for counted nuclei was set at 15–350 μm^2^ based on prior nuclei measurements. At least 20–25 images were processed for each confocal z-stack of EBs (7 EB per stage for each cell line, three experiments; total N = 84 per cell line). The percentage of EdU-labeled nuclei in relation to the total number of Hoechst-stained nuclei for each image and the average percentages for each analyzed confocal z-stack were calculated and used for the analysis of volume ratios of proliferation domains (see Supplementary Methods).

To determine the regional immunofluorescence intensity of Oct4 and Gata4 expression along the EB diameter, ImageJ/Fiji software was used to identify the associated fluorescence intensity within the identified region of interest (ROI manager). The line plots depicting diametral trends of proteins of interest in individual EB were created with the ROI/Multi plot tool. A plot of intensity values across the EB diameters in middle sections of the z-stack (for several planes) was generated in each channel. Additionally, plots of z-axis profiles of EBs were generated with the Image/Stack/Plot z-axis profile tool for analyzing intensity values through the z-axis within an ROI (100 × 100 µm) on the stacks for EBs at different stages. All measurements were carried out after checking the background intensity in each channel.

### Live-Cell Analysis of Small Molecule and Protein Diffusion Into Embryoid Bodies

The fluorescent tracers were used for the live-cell analysis of free diffusion of small molecules and proteins from culture media into the EBs and early embryos. Rodamine B isothiocyanate (RITC, MW 536; 283924, Sigma-Aldrich) was used for small molecule diffusion analysis. The fluorescent protein tracers (20 and 43 kDa) were prepared by FITC conjugating using the protocol for protein labeling (see Supplementary Methods). For diffusion analysis, the EBs and embryos were placed on the 35 mm µ-dishes or on 18 well-flat µ-slides with coverglass bottoms (Ibidi) in phenol red-free DMEM media with HEPES and L-glutamine (21063029, Gibco) supplemented with RITC or fluorescent protein tracers (final concentration 2 μg/ml) and exposed for 5, 15, 30, and 60 min. The EBs and embryos were examined under a Leica TSC SP5 confocal microscope with a temperature control system (at 37°C) using the 488 and 596 lasers and APO CS 20.0 × 0.70 IMM UV and 40.0 × 1.25 OIL UV objectives. A z-series of optical sections were done with 1–3 µm z-steps to 100–120 µm depth of specimen scanning. The RITC solution (10–15 µl**)** was injected into the inner cavity of EB10 under a stereomicroscope (Zeiss) using a glass capillary with an inner diameter 50–70 µm connected with CellTram Vario Microinjector (Eppendorf) and analyzed within 15–60 min after injection under Leica TSC SP5 microscope.

### Gene Expression Analysis

For the gene expression analysis, we collected the samples of EBs grown in FBS and KSR culture media supplemented with different combinations of LIF and RA and differently sized EBs (early and small EBs: N = 200–300 per sample for each cell number, stage, and cell line; and late and large EBs: N = 100 per sample for each cell number, stage, and cell line). Total RNAs isolated from EBs using Trizol (15596-018; Invitrogen) were treated with TURBO DNase (AM 1907; Ambion/Invitrogen) according to the manufacturer’s recommendations and the RNA yield and quality were analyzed using NanoDrop 2000 (Thermo Scientific). cDNAs were synthesized using an oligo-dT18 primer (SO131; Fermentas) and reverse transcriptase SuperScript III (18080044; Invitrogen) according to the manufacturer’s protocols. Gene expression analysis was carried out with the qRT-PCR master mix with SYBR Green stain and ROX reference dye (PK156L; Evrogen, Russia) using the Applied Biosystems 7500 Real-Time PCR System (Life Technologies). The expression levels of target genes were normalized to the expression of the reference gene hypoxanthine-guanine phosphoribosyltransferase (*Hprt*). All experiments were run in triplicate. The relative levels of target gene expression were calculated using the comparative 2-^∆∆Ct^ method (ABI Relative Quantification Study software, Applied Biosystems). Specific primers were designed using the Beacon Designer 8.0 software (Premier Biosoft) (Supplementary Table S1).

### Statistics and Data Analysis

Statistical analyses of EB growth were performed using one-way ANOVA and Student-Newman-Keuls test for post hoc analysis using the R v.3.2.3 software (http://www.r-project.org). Data presented as mean ± s.d. for three independent experiments. Analyses of EB10-to-EB1 volumetric ratios were performed using two-tailed Student’s unpaired *t*-test for normalized data collected from three experiments. The gene expression data were subjected to statistical analysis using the R v.3.2.3 software. The averaged ∆Ct values for the target gene obtained from three independent experiments (*n* = 3) were used for statistical analysis using one-way ANOVA followed by a Tukey post-hoc test. The heatmaps were constructed using a hierarchical clustering algorithm with Euclidean distances measurement based on the averaged ∆Ct values of studied genes after data scaling using the R v.3.2.3 software.

## Results

### Architecture Construction and Patterning of the EBs Formed by ESCs, EGCs, and ECCs

To standardize the 3D EB differentiation model, we used mouse ESCs, EGCs, and ECCs with the same genetic background based on the male genotype of the 129/Sv/Ev mouse strain (Bernstine et al., 1973; Nagy et al., 1993; Durcova-Hills et al., 2003). This allowed us to exclude the genotypic influence since mouse ESCs and EGCs displayed more variation in global gene expression between lines with different genotypes than between lines of various origins, while ECCs lines showed significant differences between sub-lines (Rizzino and Crowley, 1980; Moore et al., 1986; Sharova et al., 2007; Shim et al., 2008). F9 ECC line has limited spontaneous differentiation potential in conventional *in vitro* culture and after transplantation into nude mice but is capable of induced differentiation and EB formation (Gordeeva and Nikonova, 2013; Gordeeva and Khaydukov, 2017). The use of pluripotent stem and malignant teratocarcinoma cell lines allow us to focus on extracting the basic principles and specific features in the architecture construction of the EB of different cell origin (ESBs, EGBs, and ECBs).

Minimal EB size variability was achieved for all cell lines (CV EB diameters = 2.6–4.0% and 4.9–7.2% at day 1–3 and 5–10, respectively), indicating high reproducibility of model performance (Supplementary Table S2). To quantify the volumetric spherical parameters of whole EBs and their patterns at the studied stages, we first analyzed the shape descriptors, such as circularity, aspect ratio, roundness, skewness, and density, in three projections/views of the same EBs in different images. A high degree of circularity and roundness (approx. 0.9), indicating the spherical shape, was determined for the EBs at all stages. Therefore, we calculated spherical volumes of total EBs and different EB domains (see Supplementary Materials and Supplementary Table S2).

**FIGURE 1:**
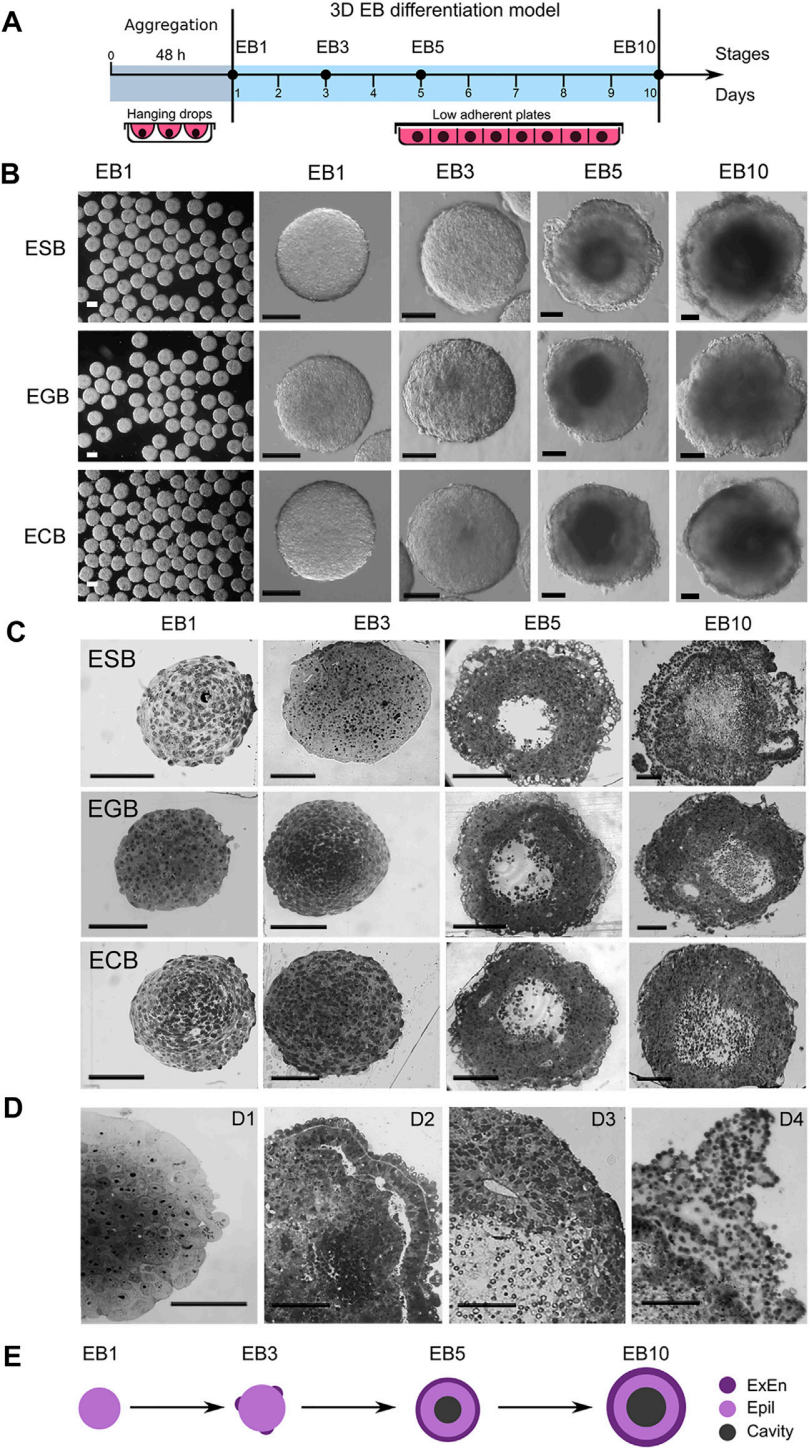
3D EB differentiation model: architectures and dynamics of ESBs, EGBs, and ECBs. **(A)** Schematic timeline for 3D EB differentiation model: EB formation in hanging drops and differentiation in suspension culture system for 10 days at EB1, EB3, EB5, and EB10 stages. **(B)** Representative images of differentiating ESB, EGBs, and ECBs with similar morphological patterns at the corresponding stages. **(C,D)** Histological sections of differentiating ESB, EGBs, and ECBs. **(C)** Similar spatiotemporal differentiation dynamics of ESB, EGBs, and ECBs and the formation of the EB archetype containing three morphologically distinct patterns. **[(D), D1–D4]** Morphogenetic events during EB differentiation: compaction **[(D),D1]** epithelization of extraembryonic endoderm (ExEn) and epiblast-like (Epi-l) cell patterns and delamination of inner cell layers **[(D),D2]** formation of internal cavities *via* the delamination and apoptosis **[(D),D3]**, and hypertrophy of ExEn immersed in the thick extracellular matrix **[(D),D4]**. **(E)** Schematic timeline for forming of the EB archetype with three distinct patterns: ExEn and Epi-l cellular patterns and inner cavity. **(B,C,D)** Scale bars: 100 μm.

We studied the architecture of ESBs, EGBs, and ECBs at four differentiation stages (EB1, EB3, EB5, and EB10) for 10 days when critical morphogenetic events occur (Figures 1A–C,E). The ESBs, EGBs, and ECBs developed similar stage-specific cellular patterns and architectures (Figures 1B,C). Early EBs (EB1 and EB3) represented uniform cell spheroids, while the EB5 and EB10 were mature cellular spheroids with spatially segregated outer cell rings of extraembryonic endoderm (ExEn) and inner epiblast-like (Epi-l) epithelialized cell layers with an internal cavity (Figures 1B,C). Histological analysis revealed the key elements of morphogenetic transformation in the EBs at the studied differentiation stages mimicking some early developmental events (Martin and Evans, 1975; Martin et al., 1977; Ducibella et al., 1982; Loebel et al., 2003; White et al., 2018): the compaction of spheroid surface similar to that of the late morulas (Figure 1D1); epithelialization of the outer and inner cell layers with the formation of a thick basement membrane resembling embryo Reichart’s membrane (Figure 1D2,D4); the formation of central cavities like the pro-amniotic cavity of an embryo (Figure 1D3); and the overgrowth of multilayer ExEn that leads to an irregular, asymmetric architecture like during the gastrulation stage (Figure 1D4).

Summarizing morphological data, we concluded that despite different cell origins, the ESBs, EGBs and ECBs develop similar EB architectures at the corresponding stages due to a similar spatial patterning and timing of morphogenetic events. During differentiation and morphogenesis, EBs of different origins establish the EB archetype homological to the embryoblast covered with ExEn at the E5.5–E6.5 stages. The EB archetype represents a radially symmetrical 3D cell structure consisting of three spatial patterns: two distinct epithelialized cell layers separated by a thick extracellular matrix membrane and an internal cavity (Figure 1E). At later stages, the deviation from the EB archetype may occur due to asymmetric overgrowths of the ExEn on the EB surface.

### Contribution of Proliferation and Cavitation to the Formation of Embryoid Body Architecture

During development, patterning is regulated through balancing cell proliferation, differentiation, and death. To understand the contribution of these processes in the EB architecture construction, first, the spatiotemporal dynamics of the cell proliferation and cavitation domains were analyzed in the EB differentiation model.

**FIGURE 2:**
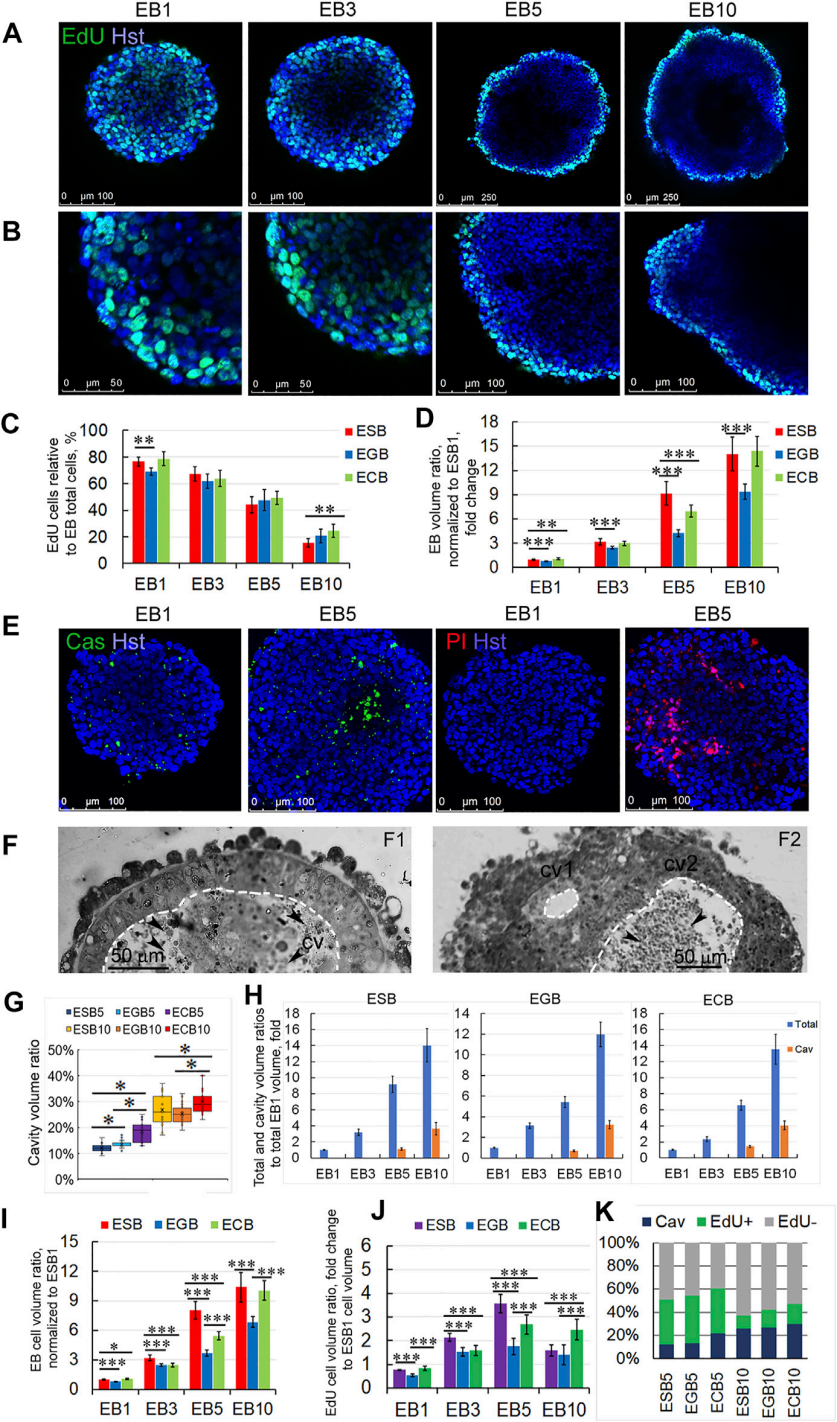
Spatiotemporal growth dynamics of ESB, EGBs, and ECBs. **(A,B)** Representative immunofluorescence confocal images of EdU-labeled cells in EBs at EB1, EB3, EB5, and EB10 stages. **(B)** Higher magnification images of A images. Nuclei were stained with Hoechst 33342 (Hst). The spatial distribution of EdU-labeled cells in EBs changes from uniform to surface during differentiation. **(C)** Proportions of the EdU-labeled cells to total cell numbers (Hst) in differentiating ESB, EGBs, and ECBs were counted using the ImageJ/Fiji cell counting tool (*n* = 7 × 4 stages for each cell line; total N = 84). The data are presented as means ± s.d., **p < 0.01, ANOVA. **(D)** EB volume growth dynamics significantly differed between ESB, EGBs, and ECBs (24 EBs/per stage; three experiments; N = 288 for each cell line). Total EB volumes at all stages were normalized to ESB1 volume. **(E)** Representative immunofluorescence confocal images of caspase activity (Cas), PI-stained necrotic cells, and total nuclei staining (Hst) in EB1 and EB5. **(F)** Histological sections showing internal EB cavities with (cv2) or without (cv2) dead cells. Cavities marked by dashed lines. **(G)** The ratios of cavity-to-total volume in EB5 and EB10 differ between cell lines (24 EBs per stage; three experiments; N = 144 for each cell line). Data presented as a boxplot with means and the 25th and 75th percentile range; whiskers indicate maximum and minimum values. **p < 0.01, ***p < 0.001, two-tailed Student’s unpaired *t*-test. **(H)** Total and cavity volume dynamics in ESB, EGBs, and ECBs normalized to corresponding EB1 volumes (24 EBs/per stage; three experiments; N = 288 for each cell line). **(I)** The ratios of cellular-to-total EB volumes differ between ESB, EGBs, and ECBs (24 EBs/per stage; three experiments; N = 288 for each cell line). **(J)** Calculated volumetric ratios of EdU-labeled cell domains to total EB volumes normalized to ESB1 increase at EB1-EB5 stages and decrease at EB5-EB10 stages (24 EBs/per stage; three experiments; N = 288 for each cell line). **(K)** The stacked barplots show the percentages of volumetric ratios of EdU-labeled and unlabeled cell domains and cavity to total EB volumes: the prevalence of the cell proliferation domain during EB1-EB5 stages and the cavitation at EB5-EB10 stages (24 EBs/per stage; three experiments; N = 288 for each cell line). The data are presented as averaged normalized means collected from three independent experiments. **(D, H,I,J)** The data at all graphs are presented as averaged means ± s.d. collected from three independent experiments. *p < 0.05, **p < 0.01, ***p < 0.001, ANOVA.

Analysis of proliferative dynamics of the ESBs, EGBs, and ECBs revealed that the cells in the S-phase of the cell cycle (EdU-labeled) uniformly distributed throughout the EB1 and EB3 volumes and predominantly in the surface cell layers in EB5 and EB10 (Figures 2A,B). The number of EdU-labeled cells gradually decreased in the range from 70–80% cells in EB1 to 15–20% cells in EB10, respectively. The number of EdU-labeled cells differed significantly between ESBs and EGBs at the EB1 stage, ESBs and ECBs at the EB10 stage (Figure 2C). Despite a decrease in the number of cells in the S-phase of the cell cycle, the total EB volumes increased 10–14 times from the EB1 to EB10 stages. Significant differences in total volumes were found between ESB, EGB, and ECB at all stages (Figure 2D). Such differences in their EB growth dynamics can have several explanations: different proliferation rates due to different cell cycle lengths for ESBs, EGBs, and ECBs at the studied stages; different dynamics of the internal cavity formation associated with cell death; and different differentiation dynamics. The most significant differences in the total volume between the cell lines (Figure 2D) were found at the EB5 stage, at which cell death and differentiation intensified (see below).

Live-cell and histological analyses revealed that cell death significantly contributes to the internal EB cavity formation (cavitation). Multiple apoptotic and necrotic cells were identified using live-cell analysis of caspase activity (Cas) and with propidium iodide (PI) and Hoechst 33342 staining in the EB5 (Figure 2E). Additionally, we identified the small cell rosettes with the lumen (Figures 1D3, 2F2), which resembled those during the pro-amniotic cavity formation (Bedzhov and Zernicka-Goetz, 2014; Orietti et al., 2021). These observations suggest that the EB central cavity may form through mixed mechanisms—apoptotic and hollowing-like (Datta et al., 2011)—in different local sites (Figure 2F). Probably, the central cavity is formed by the fusion of multiple cavities with the obligate involvement of apoptosis to remove overabundant cells during the epithelialization of inner cell layers. This assumption is consistent with previous data on EB cavitation and new data on the pro-amniotic cavity formation in double embryos (Coucouvanis and Martin, 1995; Orietti et al., 2021).

Analysis of the EB cavity volume dynamics showed an increase in the cavity to total volume ratios from 12–22% to 25–29% for EB5 and EB10, respectively (Figure 2G). The cavity volumes corresponded to 0.4–1.4- and 3.2–4.0-fold of the EB1 total volume at the EB5 and EB10 stages, respectively (Figure 2H). The differences in ratios of the cavity to total EB volume between ESBs, EGBs, and ECBs were more significant at EB5 than at the EB10 stage (Figure 2G). These data are consistent with the volumetric ratios of the pro-amniotic cavity to total embryo volume (Epi, ExEn and cavity), which were 20% and 27% for the E6.0 and E 6.5 embryos, respectively (Supplementary Figure S1).

To understand the integrated volumetric dynamics during the EB architecture construction, we calculated the increase in the EB cellular volume considering the proportion of EdU-labeled cells at each stage and the loss of EB cellular volume during the cavitation in EB5 and EB10. In general, the changes in EB cellular volumes (total volume minus the cavity volume) correlated with the gradual increase of the total volumes of ESBs, EGBs, and ECBs (Figures 2C,I). However, calculated changes in EB cellular volumes associated with the EdU-labeled cell domains indicated the increase in the volumetric proportion of proliferating cell domains from the EB1 to EB5 stages and the decrease from the EB5 to EB10 stages (Figures 2I–K). The dynamics of proliferation and cavitation domains were different between ESBs, EGBs and ECBs at most stages and the rank of gain-and-loss of EB cellular volume to EB10 stage increases in the EGBs—ESBs—ECBs row (Figures 2H–K). Thus, two opposite tendencies during the EB architecture construction were revealed: the prevalence of the cell proliferation domain during the EB1-EB5 stages and the cell death/cavitation domain during the EB5-EB10 stages (Figure 2K). ESBs, EGBs, and ECBs showed similar spatiotemporal dynamics for cell proliferation and cavitation domains but with different rate of these processes.

### Spatiotemporal Patterning and Differentiation Dynamics of ESBs, EGBs, and ECBs Consistent With the Early Developmental Trajectory

**FIGURE 3:**
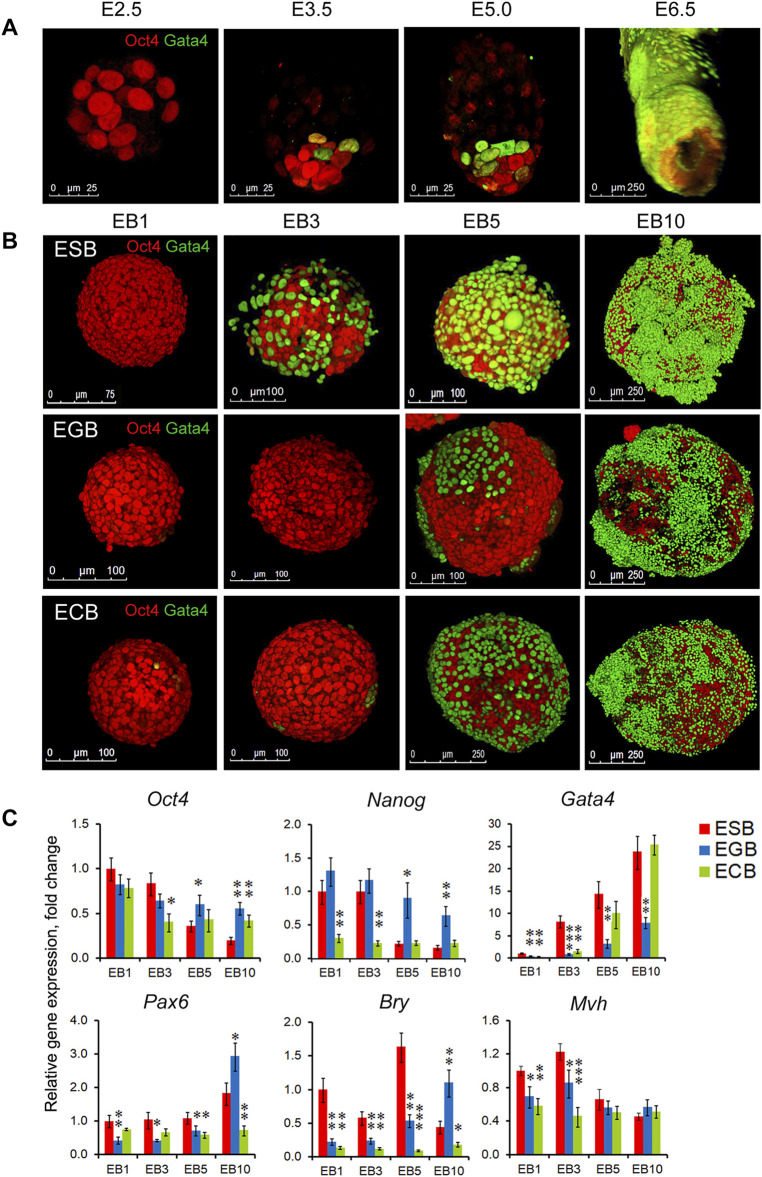
Spatiotemporal differentiation dynamics of ESB, EGBs, ECBs, and early embryos. **(A)** 3D reconstructed models of spatiotemporal patterns of the pluripotent (Oct4) and ExEn (Gata4) cells in mouse embryos at the E2.5, E3.5, E5.0, and E6.0 stages. Confocal z-stack images were processed using ImageJ/Fiji 3D Viewer tool. **(B)** 3D reconstructed models of spatiotemporal patterns of the Epi-l (Oct4) and ExEn (Gata4) cells in ESB, EGBs, and ECBs at the EB1, EB3, EB5, and EB10 stages. **(C)** Expression profiles of embryonic lineage markers in differentiating EBs: pluripotent - *Oct4* and *Nanog*, ExEn—*Gata4*, neuroectodermal—*Pax6*, mesodermal—*Bry*, germline—*Mvh*. Relative gene expression for each marker was evaluated relative to expression levels in ESB1. Data are shown as means ± s.d. from three experiments. *p < 0.05, **p < 0.01, ***p < 0.001, ANOVA.

Along with cell proliferation and cavitation dynamics, the differentiation dynamics of two EB cellular patterns also play a significant role in the EB architecture construction. To understand the consistency of earliest morphogenetic and differentiation events in the EBs and embryos, we analyzed spatiotemporal patterns for the ExEn and Epi-l cells expressing Gata4 and Oct4, respectively, at four EB differentiation stages and the E2.5, E3.5, E4.5–5.0, and E6.0 stages using 3D reconstructed models from the confocal z-stacks (Figures 3A,B). Despite the significant difference in the developmental states of cells in EBs and early embryos, morphogenetic events associated with the spatial patterning of early populations of pluripotent and ExEn cells have some similar aspects.

Early mouse embryos at the pre-implantation stages (E2.5–E5.0) progress from an 8-cell aggregate through the formation of the trophectoderm, inner cell mass, and primitive ExEn in blastocyst to the early egg cylinder stage (E6.0), consisting of the pro-amniotic cavity and five embryonic populations, trophectoderm, extraembryonic ectoderm, and visceral and parietal extraembryonic endoderm and epiblast (Figure 3A, Supplementary Movie S1, S2). Each new embryonic population arises in strict coordination with the others (Tam and Behringer, 1997; Beddington and Robertson, 1999).

The EB patterning is also associated with successive ExEn and Epi-l differentiation stages. Initially, the EB1 consists of a uniform aggregate of Oct4 expressing cells (Figure 3B, Supplementary Movie S3), whereas at the EB3 stage, single primitive ExEn cells expressing Gata4 emerge at several surface sites of a core aggregate of Oct4 expressing cells (Figure 3B, Supplementary Movies S4, S5). The timing of ExEn emerging was slightly different for ESBs, EGBs, and ECBs: the EB2-3 for the ESBs and ECBs (36–48 h) and the EB3-4 for the EGBs (48–72 h). At the EB5 stage, all three characteristic patterns develop in ESBs, EGBs, and ECBs (Figure 3B, Supplementary Movie S6). Based on the analysis of reconstructed EB models, we hypothesize that the ExEn pattern was likely formed through propagation and epiboly of individual cells rather than delaminating the entire surface cell layer in EBs. The internal cellular layers form the Epi-l pattern consisting of Oct4 expressing cells adjacent to the ExEn pattern and a cavity in the central cellular area. Finally, at the EB10 stage, the EB architecture consists of three well-formed patterns (Figure 3B, Supplementary Movie S7). Irregular ExEn outgrowths in the EB10 surface probably arise due to the absence of the trophectoderm, which is required as a spatial regulator for ExEn morphogenesis and polarity axes establishment (Tam and Behringer, 1997; Weberling and Zernicka-Goetz, 2021).

Differential expression of the early embryonic lineage markers with the most significant differences in the expression of *Nanog, Gata4, Pax6,* and *Bry* was revealed between ESBs, EGBs, and ECBs (Figure 3C). Similarly, across expression profiles of TGFβ family factors, the most significant differences were identified in the expression of *ActivinA, Lefty1, Bmp4*, and *Gdf3* (Supplementary Figure S2). Nevertheless, the gene expression profiles indicate a reproducible track of the early embryonic lineages during EB differentiation, with the greatest expression dynamics of *Gata4* and *Oct4/Nanog* and significantly lesser expression dynamics of three germ layer markers. These data are consistent with the developmental trajectory of two early embryonic lineages at the E2.5-E6.5 stages and highlight the reproducible differences in gene expression profiles and differentiation timing between ESBs, EGBs, and ECBs. However, despite common EB archetype for ESBs, EGBs, and ECBs *in vitro*, the *in vivo* differentiation patterns of EB1 and EB10 formed after transplantation into Nude mice were strictly consistent with those of undifferentiated cells of parental lines and resulted in a formation of teratomas with the derivatives of the three germ layers (ESBs and EGBs) and teratocarcinomas (ECBs) consisting undifferentiated tumor cells (Supplementary Figure S3, and Supplementary Material).

### Cell Contacts and Free Diffusion From External Environment During ESB, EGB, and ECB Differentiation and Patterning

**FIGURE 4:**
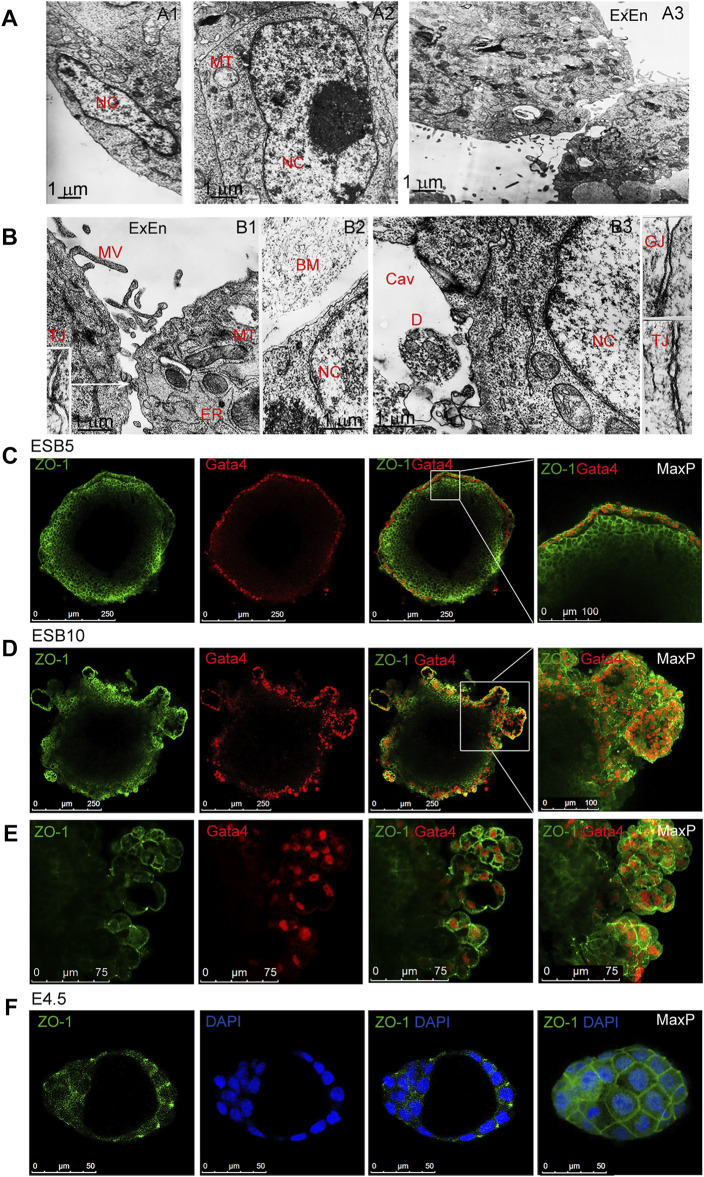
Cellular ultrastructure and contacts in differentiating EBs. **(A,B)** Electron microscopy images of outer and inner cells of EB1 **[(A),A1,A2]** and EB5 **[(A), A3], [(B),B1–B3]**. Gap (GJ) and tight (TJ) junctions between the ExEn and Epi-l cells in sidebars (140000x magnification). Nc, nucleus, MT, mitochondria, MV, microvillus; ER, endoplasmic reticulum; BM,basal membrane; Cav, inner cavity, D, cell debris. **(C,D,E)** Representative confocal images and maximal projections of ZO-1 and Gata4 double staining in ExEn and Epi-l cells of EB5 **(C)** and overgrown ExEn structures of EB10 **(D,E)**. **(F)** Representative images and maximal projections of ZO-1 and DAPI double staining in the E4.5 mouse embryo.

In the formation of EB architecture, the structuring and communication of cell layers significantly depend on intercellular contacts. Electron microscopic analysis revealed the dynamics of cell contacts, which determine the EB architecture and influence most functions of the EB cells (Figures 4A,B). In ESB1, cells of outer and inner layers had different cell and nuclei shapes while similar low nuclear-cytoplasmic ratio and cell ultrastructure with low-developed endoplasmic reticulum and multiple mitochondria (Figure 4A1,A2). In ESB5, the outer ExEn cells contained numerous microvillus and a well-developed endoplasmic reticulum secreting components of the extracellular matrix for a multilayered basement membrane that separates ExEn from the inner cells (Figure 4A3, 4B1,B2). In early EBs, only the gap junctions were detected among all cells, while both gap and tight junctions were identified in the later EBs (Figure 4B1,B3). Moreover, tight junctions were detected between the inner cells adjacent to the internal cavity of ESB5 (Figure 4B3). Immunofluorescent staining of ESBs, EGBs, and ECBs detected the ZO-1 tight junction protein in the epithelialized ExEn cells and adjacent inner Epi-l cells at the EB5 stages (Figure 4C), and afterward, in the cells of ExEn outgrowths at the EB10 stage (Figures 4D,E).

**FIGURE 5:**
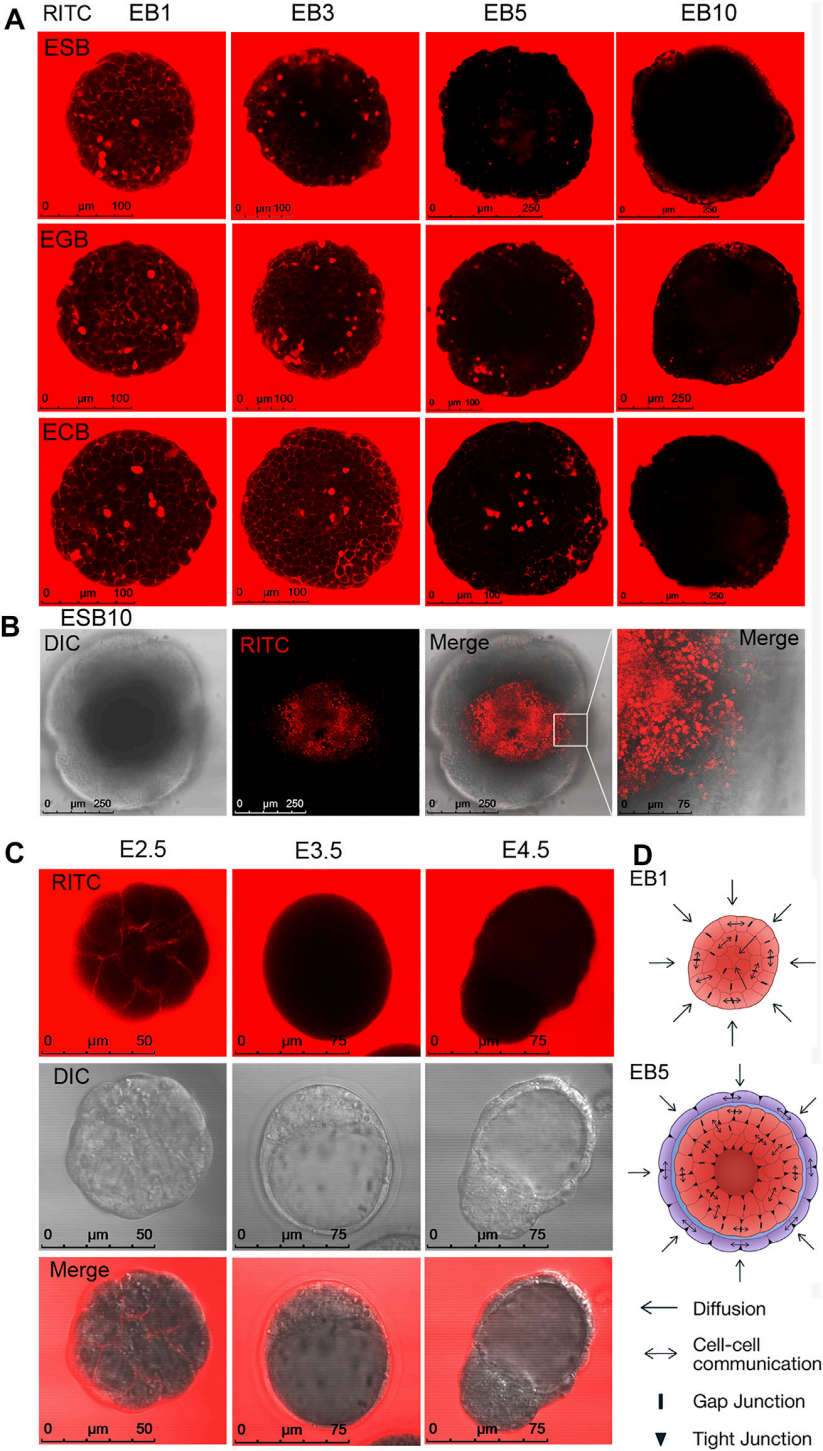
Cellular communications and small molecule diffusion in differentiating EBs and early embryos. **(A)** Representative images of live cell analysis of RITC diffusion in ESB, EGBs, and ECBs at the EB1-EB10 stages. Free RITC diffusion was detected in the intercellular space of the outer and inner cell layers of EB1 and EB3, partial diffusion in the intercellular space of EB5, and no free diffusion in EB10. **(B)** No free diffusion was detected after the RITC injection into the internal cavity of EB10 (only dead cells staining). **(C)** Free RITC diffusion was detected in the intercellular space between the blastomeres of the E2.5 morula but not in the E3.5 and E4.5 blastocysts. **(D)** The scheme of cellular communications and the diffusion of substances from the external environment modulated by intercellular contacts in EB1 and EB5. Epi-l—red, ExEn—purple, basement membrane—blue.

Live-cell confocal microscopy was used to study communications between the external environment and EB patterns. Free diffusion of the fluorescent flow tracers, fluorescent dye RITC and fluorescently labeled proteins with molecular weights of 20 and 43 kDa, was analyzed in ESBs, EGBs, and ECBs at all stages (Figure 5A, Supplementary Figures S4, S5). In accordance with the distribution of intercellular contacts, diffusion of all fluorescent tracers was detected in the intercellular space between all cells at the EB1 and EB3 stages after 5 min incubation. Intracellular diffusion of studied tracers was observed only in the dead cells (Figure 5A, Supplementary Figures S4, S5). However, at the EB5 stage, the diffusion of all tracers in the intercellular space was identified mainly between outer cell layers and sometimes between inner cells locally. Finally, at the EB10 stage, no diffusion was determined in the inner EB layers even after incubation for 1 h; although all fluorescent tracers were detected between some ExEn cells at the EB10 stage (Figure 5A, Supplementary Figures S4, S5). Moreover, no diffusion between inner cells (only dead cells staining) was detected after the RITC injection into the internal cavity of EB10 (Figure 5B). Similarly, free RITC diffusion was detected in the intercellular space between the blastomeres of the E2.5 morula but not in the E3.5 and E4.5 blastocysts, confirming the integrity of tight junctions in the trophectoderm (Figure 5C).

All these data show that during the formation of the EB archetype, there is a gradual restriction of diffusion with the external environment and between cells of different EB patterns (Figure 5D). The limitation of diffusion of small molecules and proteins in differentiating EBs occurs after forming tight junctions and a powerful basement membrane and contributes to the further EB patterning. Interestingly, the multilayered ExEn can partially compensate for the barrier function of the trophectoderm, which provides selective transport of active substances and drugs to the embryo (Marikawa and Alarcon, 2012; Eliesen et al., 2021; Gordeeva and Gordeev, 2021).

### Spatiotemporal Growth and Differentiation Dynamics of ESBs, EGBs, and ECBs Depend on External Environmental Factors

**FIGURE 6:**
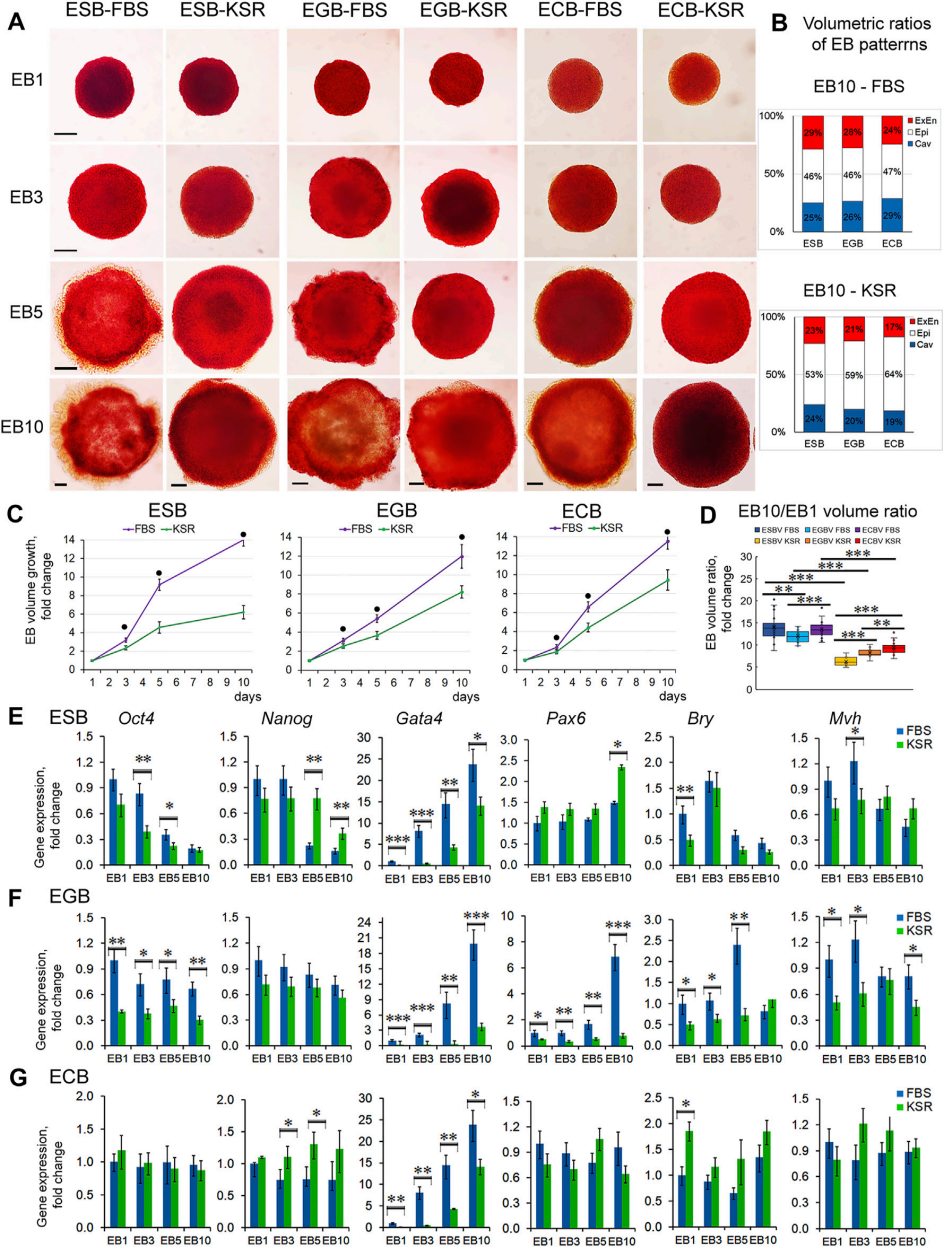
EB growth, differentiation, and patterning in different culture conditions. **(A)** Morphological analysis and ALP-staining of differentiating ESBs, EGBs, and ECBs grown in KSR- and FBS-media. **(B)** The stacked barplots indicate the percentages of volumetric ratios of ExEn, Epi-l, and cavity patterns in ESBs, EGBs, and ECBs at day 10 in the KSR- and FBS-media (24 EBs/per group; three experiments; N = 144 for each cell line). **(C)** Different EB growth dynamics in KSR- and FBS-media for all cell lines (24 EBs/per stage for each group; three experiments; N = 576 for each cell line). The data are shown as averaged means ± s.d. from three experiments. ^●^ *p < 0.001*, ANOVA. **(D)** Volumetric ratios of EB10 to EB1 for KSR- and FBS-media (24 EBs/per stage for each group, collected from tree experiments; N = 288 for each cell line). Data presented as boxplots with means and the 25th and 75th percentile range; whiskers indicate maximum and minimum values. **p < 0.01, ***p < 0.001, two-tailed Student’s unpaired *t*-test. **(E,F,G)** Expression of embryonic lineages markers in differentiating ESBs, EGBs, and ECBs grown in KSR- and FBS-media. Relative gene expression for each marker was evaluated relative to expression levels in EB1-FBS. The data are presented as means ± s.d. from three experiments. *p < 0.05, **p < 0.01, ***p < 0.001, ANOVA.

To address how the EB archetype formation is influenced by external factors/morphogens, we studied the growth and differentiation dynamics of ESBs, EGBs, and ECBs in serum (FBS) and serum-free (KSR) media with different content of growth factors. The FBS is known to contain growth factors such as IGF, FGF-2, PDGF, and TGFβ at varying concentrations in different batches, while KSR contains insulin, transferrin, and albumin at defined concentrations (Zheng et al., 2006; Chaudhry et al., 2008; Garcia-Gonzalo and Belmonte, 2008; van der Valk et al., 2010). Morphological analysis of ESBs, EGBs, and ECBs differentiating in these media revealed similar EB architectures matching the EB archetype but with different proportions and differentiation dynamics of EB patterns (Figures 6A,B). All the EBs developed thicker ExEn layers (24–29% vs. 17–23%) and larger cavities (25–29% vs. 19–24%) in FBS-media compared to KSR-media, while thicker Epi-l layers were in KSR-media (53–64% vs. 46–47%) (Figures 6A,B). These data also indicate the regular proportionality of the EB patterns: a direct relationship between the ExEn and cavity volumes and their inverse relationship with the Epi-l layer volume.

The EB growth dynamics were also significantly slower in KSR-media since the total EB10 volumes were 2.3-, 1.5-, and 1.4-fold less for ESBs, EGBs, and ECBs maintained in KSR-media than FBS-media, respectively (Figure 6C). The ratios of total volumes of EB10 to EB1 were 14, 12, and 13 vs. 6, 8, and 9 for ESBs, EGBs, and ECBs in FBS- and in KSR-media, respectively (Figure 6C). Moreover, the differences in the EB growth dynamics are retained in both KSR-media and FBS-media with various supplement combinations of LIF, which promotes self-renewal of undifferentiated pluripotent cells and retinoic acid (RA), which induces differentiation (Supplementary Figures S6). The down-regulating growth effects of RA were revealed for EB5s of all cell lines, while LIF enhanced the growth of ESBs and EGBs only. Expectedly, LIF and RA combination annihilated the growth effects in ESBs and EGBs (Supplementary Figures S6A,B).

Different differentiation dynamics were also confirmed by quantitative gene expression analysis of embryonic lineage markers in ESBs, EGBs, and ECBs grown in KSR-media and FBS-media (Figures 6E–G). The lesser differences in the marker gene expression between EBs cultivated in KSR- and FBS-media were characteristic for ECBs, and the largest for EGBs. For all EBs, the expression dynamics of *Gata4* were most different, while the *Pax6, Bry*, and *Mvh* expressions faintly differed in ESBs and ECBs, but significantly differed in EGBs. Cell line- and stage-specific differences in the *Oct4* and *Nanog* expression were found but were most pronounced at the EB5 stage. Opposite expression dynamics of *Oct4* and *Gata4* were identified in EB5 treated with the LIF and RA: up-regulated expression of *Oct4* after LIF treatment and *Gata4* after RA treatment, compared with untreated control (Supplementary Figures S5C,D).

These data suggest that variable dynamics of marker gene expression in spontaneously differentiating ESBs, EGBs, and ECBs may be caused by their different epigenetic states and mutations in ECCs. The epigenetic and genetic variations can lead to different responses to growth factors eliciting embryonic lineages in different environments (Ortmann and Vallier, 2017). Although, even in the absence of differentiation-promoting factors in KSR-media, all EBs can differentiate into the earliest ExEn lineage by inducing the *Gata4* expression. Simultaneously, gene markers of later lineages (*Pax6, Bry,* and *Mvh*) were expressed at a low level in all EBs in both media but significantly lower in the KSR-media. Similarly, the *Oct4* and *Nanog* expression dynamics were significantly less in the KSR medium. Under differentiation-promoting conditions with RA treatment, the *Oct4* and *Gata4* expressions were less different, both in all EBs5 (Supplementary Figures S6) and 2D culture systems (Sharova et al., 2007; Gordeeva et al., 2019). All these results indicate the critical role of the levels of differentiation factors/morphogens in embryonic lineage induction. Consequently, the growth and differentiation dynamics of ESBs, EGBs, and ECBs significantly depend on both external environmental factors and cell states. The cellular composition and proportions of EB cellular patterns can be regulated by a specific combination of growth and differentiation factors.

### Spatiotemporal Growth and Differentiation Dynamics of ESBs, EGBs and ECBs Are Independent on Cell Numbers and EB Size

**FIGURE 7:**
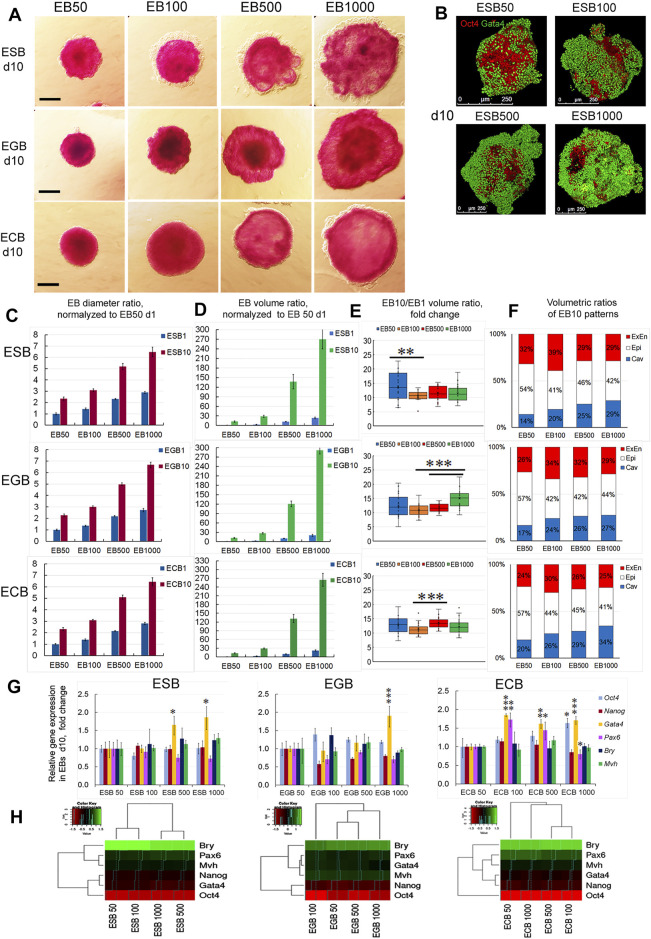
Growth, differentiation, and patterning of EBs formed by different cell numbers. **(A)** ALP-staining of ESBs, EGBs, and ECBs formed by 50,100, 500, and 1,000 cells at the 10th day of differentiation. **(B)** 3D reconstructed models of spatial patterns of the Oct4 and Gata4 expressing cells in ESBs formed by different cell numbers at day 10. **(C, D)** Diameter and volume ratios for differently sized ESBs, EGBs, and ECBs normalized to respective EB50 at day1 (24 EBs/per group for each cell line; three experiments; N = 288 for each cell line), data presented as averaged means ± s.d. **(E)** Volumetric ratios of EB10 to EB1 for differently sized ESBs, EGBs, and ECBs day1 (24 EBs/per group for each cell line; three experiments; N = 576 for each cell line). Data presented as boxplots with means and the 25th and 75th percentile range; whiskers indicate maximum and minimum values. **p < 0.01, ***p < 0.001, two-tailed Student’s unpaired *t*-test. **(F)** The stacked barplots indicate the percentages of normalized volumetric ratios of ExEn, Epi-l, and cavity patterns for differently sized ESBs, EGBs, and ECBs at day 10 (24 EBs/per group for each cell line; three experiments; N = 288 for each cell line) **(G,H)** Expression of embryonic lineages markers **(G)** and profile clustering **(H)** for differently sized ESBs, EGBs, and ECBs at day10. Relative gene expression for each marker was evaluated relative to expression levels in EB50 for each cell line. Heatmaps and hierarchical dendrograms show the gene expression profile clustering for differently sized EB of each cell line at day10. The data are presented as means ± s.d. from three experiments. *p < 0.05, **p < 0.01, ***p < 0.001, ANOVA.

To understand size control mechanisms in the EB differentiation model, we investigated spatiotemporal growth and differentiation dynamics of differently sized EBs formed by various cell numbers. Analysis of differentiating ESBs, EGBs, and ECBs formed by 50, 100, 500, and 1,000 cells revealed no differences in their temporal dynamics and the final architectures at the EB10 stage, despite their significantly different sizes (Figures 7A–E). The time of appearence and spreading the ExEn and cavity occurred at an almost similar pace in all EBs. However, EB50 for all cell lines displayed a smaller ExEn cell pattern and internal cavity (Figure 7F), and therefore, their volumetric proportions differed from other sized EBs. The differences in the EB50 patterns’ proportions may be due to the greatest size variability during the formation and the slower cavitation.

Growth dynamics of differently sized EBs was proportional to the initial EB1 volume since the volumetric ratios of EB10 to EB1 were in the similar ranges of 10–14, 11–15, and 11–13 for ESBs, EGBs, and ECBs, respectively. Differences in these values between EB50, EB100, EB500, and EB1000 were mostly insignificant within each cell line (Figures 7C–E). However, differences in growth rate between some EB groups of each line were found and need further studies to identify possible technical and biological causes.

Analysis of embryonic lineage marker expression in the ESBs, EGBs, and ECBs formed by 50–1,000 cells on day 10 detected significant differences in the *Gata4* expression for most EB500 and EB1000, although the differences in expression levels were no more than two times (Figure 7G). Among the ECBs, differences in the *Pax6* expression were also found between EB100 and EB500 compared to EB50 (Figure 7G). Simultaneously, the expression of *Oct4* and *Nanog* did not differ significantly between most differently sized EBs for all cell lines (Figure 7G). Hierarchical clustering of marker expression profiles in differently sized EBs reveals no general trends since the most distinct and similar EB groups were specific for each cell line (Figure 7H).

Thus, ESBs, EGBs, and ECBs can maintain the invariant proportions of the archetypal EB structure and do not adjust the growth and differentiation rate relative to their size and initial cell numbers. Whilst experimentally enlarged or reduced early mouse embryos can regulate their size during the pre-gastrulation stages (E5.5-E5.75) and develop into normal-sized pups (Tarkowski, 1963; Buehr and McLaren, 1974; Lewis and Rossant, 1982; Orietti et al., 2021). These data suggest that EBs can scale their spatiotemporal patterns during architecture construction.

### Pattern Regulating and Scaling in the EB Differentiation Model

**FIGURE 8:**
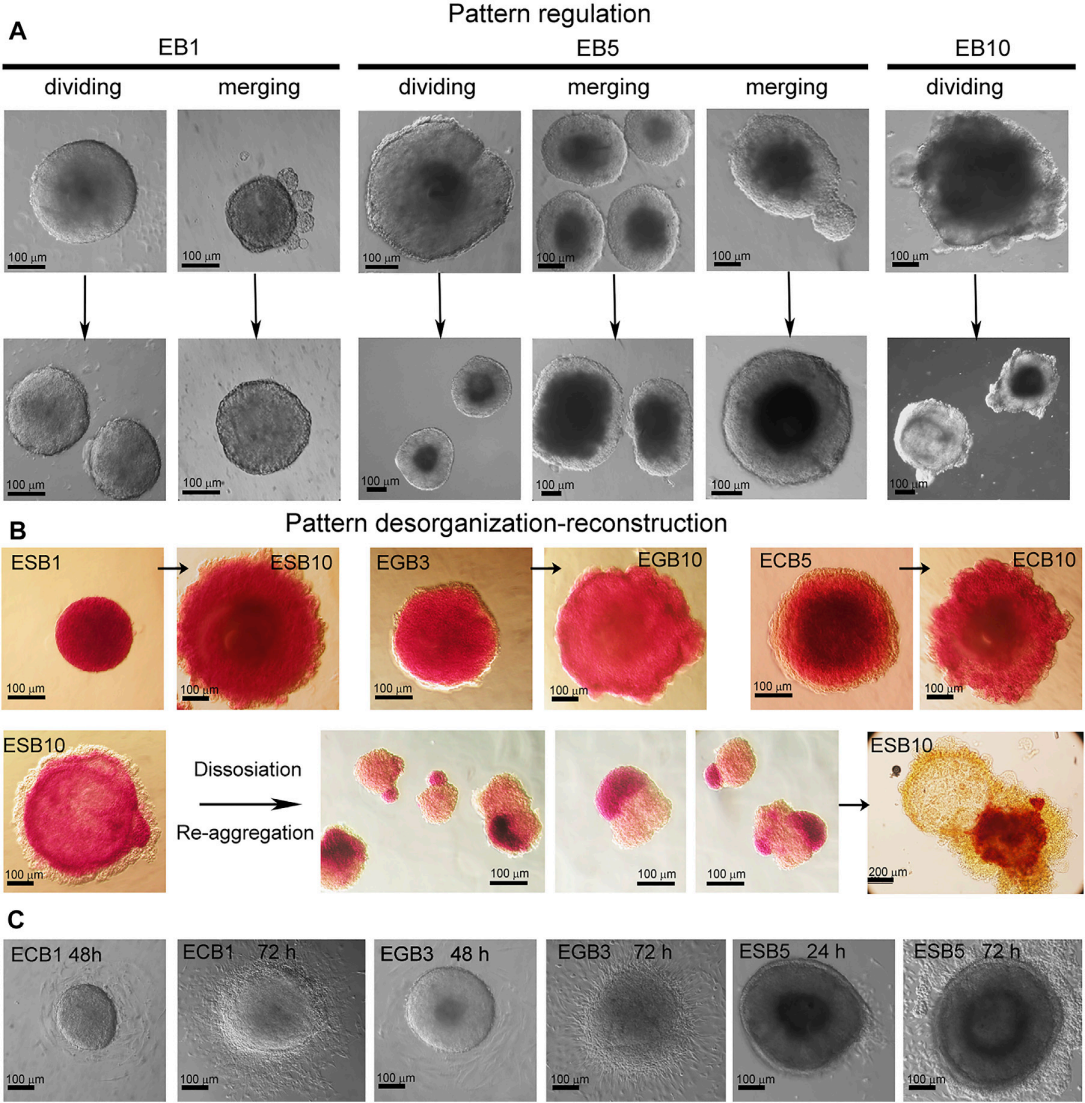
EB pattern regulation after dividing, merging, and disorganization. **(A)** Representative images of pattern regulation in the EB1, EB5, and EB10 after dividing and merging. After merging or dividing, the resulting EBs restored their patterns and architecture and continued further differentiation in all cases (100%) (10 EBs/per stage per cell line; three experiments; N = 120 for each cell line). **(B)** Pattern disorganization-reconstruction by dissociating EBs and re-aggregating cell suspension for 72 h (5 EBs/per stage for each cell line; three experiments; N = 60 for each cell line). Dissociated EB10 cells (100 cells/per drop) *de novo* self-organize and sort within the aggregate into arbitrary spatial arranged cellular domains consisting of homogeneous populations of ExEn and Epil cells. During further differentiation, all three typical EB patterns are restored but with varying degrees of symmetry. Cavities are formed within the Epil and sometimes ExEn domains. ALP activity was detected in EBs and reconstructed cell aggregates with asymmetrical patterns. **(C)** Representative images of spherical shape loss and pattern disorganization after the attachment of EB1, EB3, and EB5 to cell culture dishes at 24–72 h. EB1-5 cells can attach to the dish surface and migrate, while EB10 can not.

Given our finding that the morphogenesis of the EB archetype is independent of cell numbers forming EBs (Figure 7), we next analyzed the possibility of the pattern regulation after experimental dividing, merging, and deconstruction-reconstruction of ESBs, EGBs, and ECBs. First, we examined the EB pattern regulation after dividing or merging (Figure 8A). These experiments demonstrated that early EB1 and later EB5 and EB10 can restore their patterns and continue further differentiation and patterning after merging or dividing. In the case of merging EBs at different differentiation stages (EB1-EB3 and EB1-EB5), the resulting patterns and architecture corresponded to a later stage. Early EBs (EB1-EB3) easily regained their spherical shape for 2–4 h, while EB5 restored shape and patterns for 24–48 h after merging or dividing. Although the EB10s never merged, dividing EB10 resulted in two EB10s with partially restored and asymmetric patterns for 48 h. Thus, these results showed that, like in the EBs formed by different numbers of cells, overall sizes of merging or dividing EBs are not regulated, but EB patterns and architecture are restored during the following days of differentiation.

We next examined whether EB patterns are regulated after dissociating ESBs, EGBs, and ECBs at different stages. The experiments with pattern disorganization-reconstruction by EB dissociation and re-aggregation of cell suspension (100 cells in hanging drops for 72 h) revealed *de novo* self-assembly and architecture construction for the EB1, EB3, and EB5 (Figure 8B). In contrast, dissociated EB10 cells re-aggregated and sorted within the aggregate into arbitrary spatial arranged cellular domains consisting of homogeneous ExEn and Epi-l cell populations (Figure 8B). During further differentiation, the Epi-l domain becomes internal due to the spreading of ExEn cells over its surface, and then cavities are formed within the Epi-l and sometimes ExEn domains. Thus, all three archetypal EB patterns are restored but with varying symmetry degrees. ESBs, EGBs, and ECBs showed similar features in restoring their patterns during these experiments.

Together, these results demonstrate that EB1, EB3, and EB5 easily regain their spherical shape and patterns, as their cellular fates are not fully determined. Additionally, EB1-EB5 cells retain their migration ability, as they easily adhere and migrate over the culture dish surface (Figure 8C). In EB10, no fusion, adherence, and reorganization of shape and cellular patterns proceeded, probably due to the fixed spatial position of differentiated cells through specific contacts. However, after EB10 dissociation, ExEn and Epi-l cell migration significantly contributes to *de novo* self-organizing EB structure. These results are consistent with previous studies of the capacity to re-establish experimentally disorganized patterns through cell sorting and spreading based on differential adhesion (Armstrong, 1989; Maeda et al., 2007; Choi et al., 2016; Revell et al., 2019; Scalise et al., 2021).

## Discussion

In this study, we explored a highly reproducible experimental platform, the 3D EB differentiation model, which generates the EB archetype mimicking architectures and developmental trajectories of the early embryos (Beddington and Robertson, 1999; Rivera-Pérez and Hadjantonakis, 2015; White et al., 2018; Sozen et al., 2019). The EB archetype, consisting of three individual morphological patterns—ExEn, Epi-l, and internal cavity—was established by the ESCs, EGCs, and ECCs without the involvement of the trophectoderm lineage. This suggests the possible independence in the architecture construction for the earliest epiblast covered with extraembryonic endoderm from the trophoblast, which selectively isolates the embryo from external influences and plays a significant role in the formation of the polarity axes of the early embryo (Smith, 1980; Marikawa and Alarcon, 2012; Weberling and Zernicka-Goetz, 2021). Therefore, the EB archetype represents a radially symmetric 3D cell structure without the polarity axes. Interestingly, even in the ETX-embryos containing trophectodermal cells, only the proximal-distal axis, but not the anteroposterior axis, is set in them (Sozen et al., 2019). Moreover, the appropriate embryonic patterns were identified in only 20% and 30% of formed ET- and ETX-embryos, respectively (Sozen et al., 2018, 2019).

All stages of the EB architecture formation are closely related to the symmetry breaking. Although, at all differentiation stages, EBs remain predominantly radially symmetric 3D cellular structures at a large-scale level, the formation of all three patterns is associated with symmetry-breaking events at a smaller local scale (Figures 1B,D, 3B). As mentioned above, the first symmetry-breaking event is associated with the appearance of single ExEn cells and the initiation of external cell patterning. Subsequent symmetry-breaking events are related to the establishing apical-basal polarity in the ExEn and Epi-l cell layers, which lead to the formation of two functionally distinct types of epithelium (Figures 1C, 4). Local symmetry breaking continues with further differentiation of these cell patterns, resulting in asymmetric outgrowths in the ExEn pattern and cavity formation in the Epi-l pattern (Figures 1C,D, 3B). It can be assumed that all these symmetry-breaking events can be triggered by changes in the morphogens’ diffusion from the external environment (Figure 5, Supplementary Figure S4) and changes in the expression of TGFβ factors during differentiation (Supplementary Figure S2). Such changes may lead to establishing local morphogens’ gradients within these patterns. However, despite the successive symmetry-breaking events during EB patterning, no morphological signs of the presumptive polarity axes were observed in mature EB10 (Figures 1B,C). The lack of spatial boundaries formed by the trophoblast and the blastocyst cavity may explain the disturbance in establishing the EB proximal-distal axis, but not the anterior-posterior axis. Therefore, further research is needed to identify the mechanisms that may underlay the polarity axes formation in the EB differentiation model.

Analyzing the contribution of cell proliferation, death, differentiation, patterning, and intercellular communication in the EB architecture construction, we found changing trends in the ratio of the cell proliferation and death domains (Figure 2) and a gradual restriction of diffusion from the external environment and between cellular layers (Figure 5, Supplementary Figures S4, S5). Presumably, the restricted communication between cellular patterns through diffusion modulating (tight junctions and thick basement membrane) can drive further pattern development contributing to internal morphogens’ gradient and the mechanical stress for epithelialized cell layers. On the one hand, the extensive contact with the external environment can lead to hypertrophy of the ExEn pattern (Figures 1D4, 3B, 7B). On the other hand, the restriction of free diffusion of growth factors/morphogens from the culture media to EB5-EB10 inner cellular layers (Figure 5, Supplementary Figures S4, S5) can result to the prevalence of cell death/cavitation domain over proliferation domain (Figure 2K). With EB cavity patterning, the Epi-1 cells adjacent to the basement membrane preferentially survive and proliferate (Figures 2A,F), as previously shown for early embryos and ETX-embryos (Bedzhov and Zernicka-Goetz, 2014; Harrison et al., 2017). To promote more advanced EB stages, the EB cavitation and ExEn regulation mechanisms require further investigation.

**FIGURE 9:**
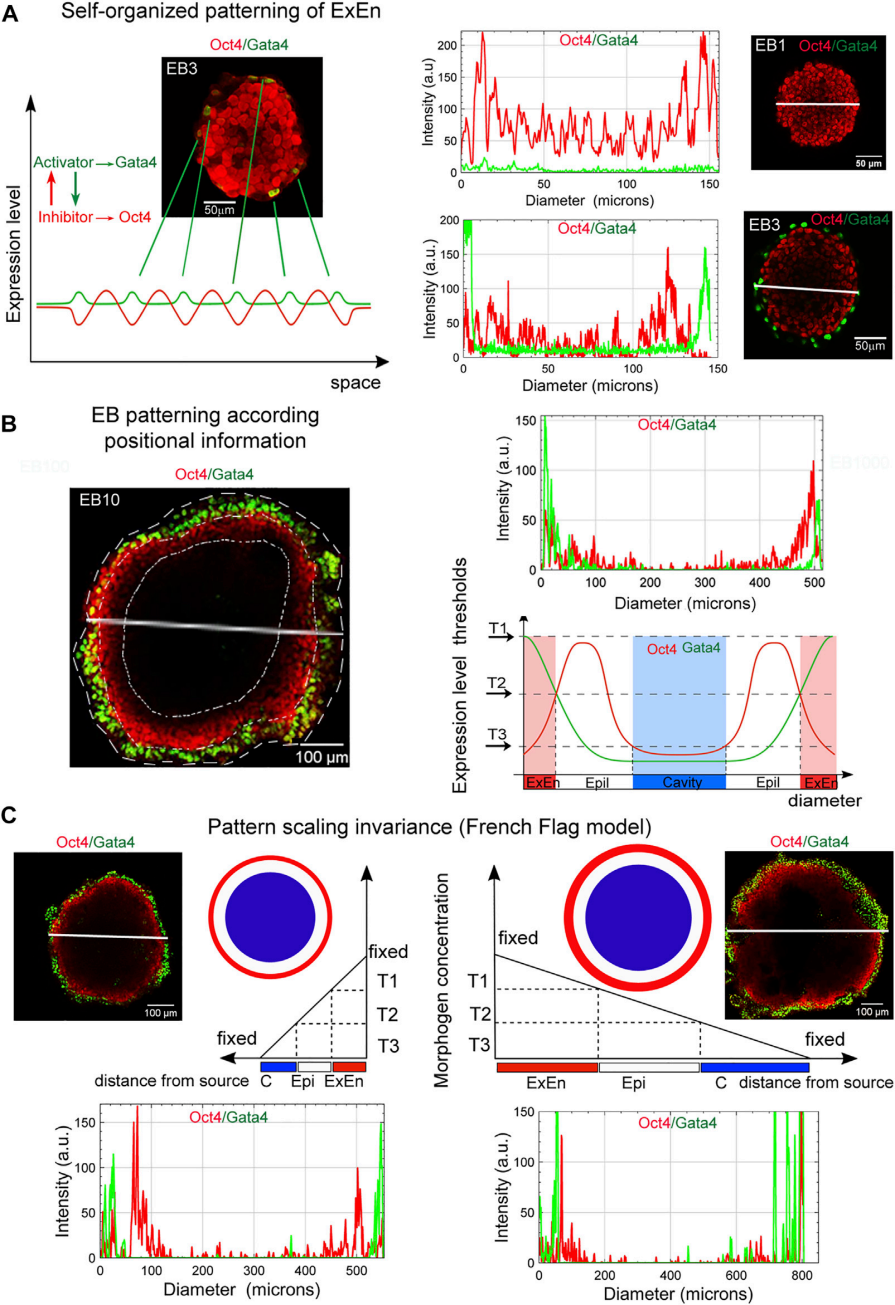
Basic principles in EB patterning and scaling. **(A)** The ExEn pattern in early EBs can emerge due to random changes in the Gata4 and Oct4 expression in surface cells, which may correspond to the RD model of self-organizing patterns in homogeneous systems. Representative plots of spatial trends for the intensity of the Oct4 and Gata4 expression across diameters (marked by white lines) in respective images of EB1 and EB3 demonstrate changes in Gata4 expression in surface cell layers. Similar trends in signal intensity changes were observed when analyzing the along the z-axis (see Supplementary Figure S7A,B). **(B)** Three morphological patterns in EBs, ExEn, Epi-1, and the cavity, can presumably form in accordance with the position of the cell layers under conditions of a morphogen concentration gradient and free diffusion from the external environment. The EB archetype patterning may be consistent with the French Flag paradigm. The highest morphogens’ concentrations (T1) can induce the ExEn pattern (Gata4^high^/Oct4^low^), moderate concentrations (T2) can contribute to maintaining the Epi-l pattern (Oct4^high^/Gata4^low^), and low concentrations (T3) can trigger apoptosis and cavitation (Oct4^low^/Gata4^low^). A representative plot of spatial trends for the intensity of expression of Oct4 and Gata4 across EB10 diameter (marked by a white line) shows the distribution of expression levels of these marker proteins in accordance with the EB patterns depicted in the diagram below. **(C)** A scheme showing the expected EB pattern scaling invariance model (French flag model for volumetric scaling). Pattern scaling in differently sized EBs may be consistent with the models of scaling by specific boundary conditions and a diffusion modulator. T1, T2, and T3 threshold levels of morphogens (constant concentration in cell culture media) can define similar ExEn, Epi-l, and cavity proportions in small and large EBs (see Figure 7F). Representative plots of spatial trends for the intensity of Oct4 and Gata4 expression in EB100 and EB1000 indicate the similar distributions of expression of these marker proteins across diameters in accordance with the EB patterns depicted in the pictures above. Similar trends in signal intensity changes are observed along the z-axis scanning of EB100 and EB1000 (Supplementary Figure S7C,D). Distributions of Gata4 and Oct4 staining intensity along the z-axis indicate ExEn and Epi-l patterns of different sizes in EB100 and EB1000.

Given our finding that the formation of the EB archetype during ESB, EGB, and ECB morphogenesis can recapitulate some aspects of the early development, we next considered whether the basic principles of RD and PI morphogenetic concepts could be involved in the self-organization, patterning, and scaling in the 3D EB differentiation model. According to our observations, the onset of EB patterning is associated with the appearance of single ExEn cells at several surface locations after LIF withdrawal in culture media (Figure 3B, Supplementary Movie S4). We assumed that the ExEn cell type may appear due to random changes in the Oct4 and Gata4 expression (activator-inhibitor TFs) in surface cells (Figure 9A), which are stimulated through several intermediate steps in Erk/Mek and TGFβ family signaling pathways (Burdon et al., 1999; Fujikura et al., 2002; Frankenberg et al., 2011).

The triggering of the differentiation of pluripotent cells due to the random changes of transcription factor expression in single cells was previously examined experimentally (Plachta et al., 2011; Pantazis and Bollenbach, 2012) and by theoretical modeling (Halley et al., 2009, 2012; Greaves et al., 2017). The developed conceptual model proposes that stochastic gene expression within a stem cell gene regulatory network self-organizes to a critical-like state, which primes various transcriptional programs associated with different cell lineages. This model is consistent with the concept of a self-organizing pattern in the RD system, which postulates that a new pattern can arise during chemical morphogenesis after interactions of two homogeneously distributed substances (morphogenes), which generate stable periodic pattern even from a random or uniform initial condition. These patterns represent local differences in the concentrations of the two substances (Turing, 1952).

Based on our observations, we hypothesized that the PI interpretation by EB cells might also determine cell fate during EB patterning. Thus, the external EB cell layer has the largest contact area with the external environment and can be exposed to the maximum concentrations of growth factors/morphogens. Hence, they are probably the first to respond to the stimulation (Figure 9A). We have detected the ExEn pattern only on the surface of EBs, even in media with different growth factors’ contents (Figures 3B, 6). Therefore, we believe that the outer ExEn pattern is most likely formed due to the proliferation and spread of initiator cells over the surface until an enclosed cell layer is formed (Figure 3B, Supplementary movies S3, S5, S6). However, this assumption needs to be confirmed by live-cell analysis. Simultaneously, EB inner cells retained a low-differentiated state since they continued to express Oct4 and ALP (Figures 3B, 5, 6). Two possibilities can explain this: firstly, by the remoteness from the source of differentiation factors, and secondly, by establishing tight junctions and the basement membrane limiting diffusion in late EBs. The EB patterning is fundamentally different from the 2D gastruloid and peri-gastrulation-like patterning for hESC colonies with simultaneously arising radial cell layers of lineage progenitors (Warmflash et al., 2014; Deglincerti et al., 2016; Etoc et al., 2016; Tewary et al., 2017). These differences may reflect mouse and human specific features of embryonic stem cell patterning and the differences between 2D and 3D models.

Next, we considered whether the EB patterning is consistent with the classic French flag paradigm (Wolpert, 1981). The PI conceptual model describes embryonic patterning in terms of a coordinate system determining polarity as the direction in which positional information is specified or measured (Wolpert, 1981; Umulis and Othmer, 2013; Green and Sharpe, 2015; Čapek and Müller, 2019). In the French flag paradigm, the ability of the system to adjust the pattern when parts are removed or added and to demonstrate size invariance is related to the ability of cells to change positional information and interpret it. In the EB archetype, the cell fate in each EB pattern presumably may be determined by the PI of cell layers as a distance from the morphogens’ source (Figures 9B,C). According to the PI concept, these three patterns might be formed due to the morphogen concentration gradient arising *via* free diffusion from the external environment. The spatial distribution gradient of soluble growth factors, nutrients, and oxygen may be formed due to uneven diffusive mass transfer within EBs. The modeling and direct measurement of oxygen and cytokine concentrations suggest that the size of EB strongly affects the threshold at which the system becomes mass transfer limited (Wartenberg et al., 2001; Van Winkle et al., 2012). If such gradients are formed, the highest threshold concentrations of morphogens can give rise to the ExEn pattern (Gata4^high^/Oct4^low^), moderate concentrations can maintain the Epi-l pattern (Oct4^high^/Gata4^low^), and low threshold concentrations of morphogens can trigger apoptosis and cavitation (Oct4^low^/Gata4^low^) (Figure 9B). The differentiation rate and the size of the ExEn pattern can depend on the concentration of growth factors/morphogens from the external environment since thicker ExEn patterns and higher *Gata4* expression were detected in EBs cultured in FBS-media compared to KSR-media (Figures 6A,B,E-G). Notably, the final ExEn pattern formation does not depend on the free EB surface area and continues in ExEn outgrowths hereafter (Figure 3B). After establishing all three patterns, the ExEn and Epi-l patterns continue their development, possibly following a new morphogen gradient.

Analyzing the scale invariance in the EB differentiation model (Figures 7, 8), we suggested that pattern scaling in differently sized EBs may be explained *via* the models of scaling by specific boundary conditions and diffusion modulators (Čapek and Müller, 2019). It can be assumed that the ExEn pattern in small and large EBs is formed by the different numbers of initiator cells under free diffusion and maximum morphogen concentrations (Figure 9C). However, with fixed boundary conditions (growth factor concentrations and EB surface area) and emerging diffusion modulators (tight junctions and the basement membrane), morphogens’ gradient can probably remain proportional to the EB surface area and volume. Additionally, the concentrations of secreted morphogens and extracellular matrix proteins may remain proportional to the number of ExEn cells.

Previous studies and our current study have shown that EB differentiation and patterning are highly dependent on the growth factors/morphogens from the culture media with FBS and KSR and their endogenous gene expression of TGFβ family factors (Dang et al., 2002; Bauwens et al., 2008; ten Berge et al., 2008; Bratt-Leal et al., 2009; Giobbe et al., 2012; Chhabra et al., 2019). Our study found that the growth factors/morphogens’ concentration in the culture media does not affect the EB archetype structure, even for the ECBs, whereas it affects growth and differentiation dynamics, cellular composition, and patterns’ proportions (Figure 6, Supplementary Figures S5). All EBs formed more voluminous ExEn and cavity patterns but less voluminous Epi-1 patterns in the FBS medium than in the KSR medium. Importantly, the ESBs, EGBs, and ECBs with different expression levels of TGFβ factors (Supplementary Figures S2) form similar EB architectures (Figures 1B,C, 3B), i.e., strived for the unified EB archetype. All these data can serve as a basis for experimental engineering and theoretical modeling of EB patterns through modulation by various morphogens/signaling pathways, like previously developed peri-gastrulation-like patterning models for hESC colonies (Warmflash et al., 2014; Deglincerti et al., 2016; Etoc et al., 2016; Tewary et al., 2017). These studies demonstrated how the modulation of BMP4 concentration in culture medium induces self-organization of pSMAD1 gradient in hESC colonies, which stimulates a radial pre-patterning cell fates with the trophectoderm-like fate (CDX2) at the colony edge, followed by the endoderm-like (SOX17) and primitive streak-like (BRA) regions, and the ectoderm-like (SOX2) region at the colony center (Etoc et al., 2016; Tewary et al., 2017). The cell fate acquisition depends on both the pSMAD1 signaling strength and duration of induction and is consistent with the positional information of cells. The formation of such a pre-patterning may depend on the colony size and cell density in the colonies that correlate with the “edge-sensing” basolateral or apical distribution of BMP receptors in the hESC membranes and can be adjusted by the morphogen concentration according to the colony size. Interestingly, the trophectoderm-like fate pre-pattern identified at the hESC colony edge never forms in mouse EBs with an external ExEn pattern. It should be considered that both the EB and hPSC colony patterning depend on the exogenous and endogenous morphogens and coordinated interaction of multiple signaling pathways, which need further investigations using the principles of RD and PI concepts.

Taking together our considerations and assumptions, we can conclude that both RD and PI concepts may be parallel involved in the EB archetype formation. RD may initiate the self-organizing ExEn patterning consistent with the PI of EB surface cells. The subsequent formation of ExEn, Epi-l, and cavity patterns could also be determined by their PI, as the distance of cell layers from the external morphogens’ source, and the morphogens’ concentration gradient, which can be formed in accordance with RD. However, the upstream or downstream mode of RD and PI at specific stages of the EB patterning is challenging to define, and further experimental research is needed.

In conclusion, the developed 3D EB differentiation model of self-organized patterning and architecture construction provides an opportunity for experimental and computational modeling and analyzing the mechanisms of cellular diversity and spatiotemporal patterns under the morphogen influence. The presented model is free from ethical concerns and can be used for drug discovery and toxicological research. This model is physiologically approximated to *in vivo* conditions and imitates some early developmental events. Our investigative approaches to studying the formation of EB archetypal structure can also be helpful for organoid and cancer spheroid research.

## Data Availability

The original contributions presented in the study are included in the article/Supplementary Material, further inquiries can be directed to the corresponding author.
