# Supplementary material for: Archetypal Architecture Construction, Patterning, and Scaling Invariance in a 3D Embryoid Body Differentiation Model
Source: Front Cell Dev Biol. 2022 Apr 27;10:852071. doi: 10.3389/fcell.2022.852071 (PMC9091174; doi:10.3389/fcell.2022.852071)
Supplement: Supplementary file 2 [file DataSheet1.PDF]

Supplementary Material

Supplementary Materials and Methods

EB shape descriptors and measurements

To evaluate EB growth dynamics and patterning, the EBs (n = 24 per group in each experimental series) cultivated in 12- or 96-well plates with a low-attachment surface (Greiner Bio-One International GmbH and Nunclon Sphera; Nuclon/Thermofisher Scientific) and imaged at all the studied stages (EB1,3,510). To prevent EB adhesion to the surface and loss of spherical shape, the plates were continuously shacked during the cultivation. The EB shape was monitored and EBs that lost their spherical shape due to adhesion to the surface were excluded from the experiments. EB images were captured using an Olympus CK40 inverted microscope equipped with a Camedia C-4040 camera (Olympus). Image processing and determining the EB shape descriptors such as an aspect ratio, circularity, roundness, skewness, and solidity were performed using ImageJ/Fiji software (https://imagej.nih.gov/ij/) with prior set up absolute scale calibration. For assessing the spherical shape, the mentioned shape descriptors were determined in several projections of the same EBs at different images for the EBs1, EBs5, and EBs10 stages. Based on the image analysis data confirming the spherical shape of EBs at the studied stages, since the aspect ratio, circularity, roundness, and solidity were apr. 0.9, the calculation of the EB volumes and their patterns was performed as for spherical objects using averaged values of diameter and length (see Table S2). Statistical analysis of these descriptors for the total volume and cavity volume in EBs of different stages was performed using ANOVA, with a significance level of $P<0.05$. The P values are shown at the end of Table S2.

Diameter measuring and calculating volumes of EBs and EB patterns

Total EB diameters, cavity diameters and heights of ExEn layers were measured in two to six directions for average calculation using ImageJ/Fiji software with prior set up absolute scale calibration. For each EB at each stage, total EB volume, volumetric pattern sizes and their ratios were calculated using Excel and the following formulas for the sphere volume calculation.

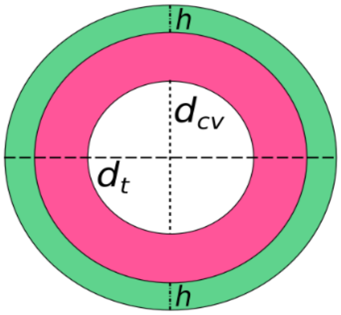

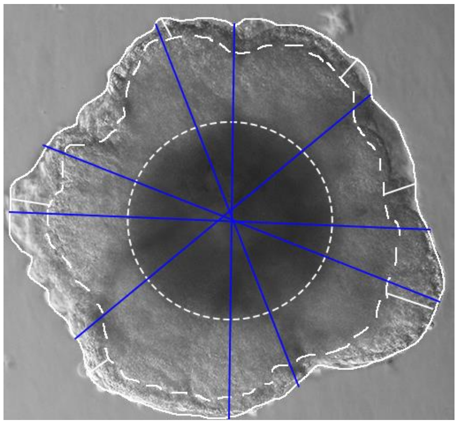

Total EB volume (V_t):

$V_t = \frac{\pi}{6} d_t^3$ d_t – average EB diameter

Total EB volume (V_t) was also defined as sum of volumes of three EB patterns:

$V_t = V_{ex} + V_{e_P} + V_{cv}$

V_{ex} – ExEn pattern volume

V_{ep} – Epi-l pattern volume

V_{cv} – cavity volume

Cavity volume was calculated as:

$V_{cv} = \frac{\pi}{6} d_{cv}^3$, where d_{cv} – average cavity diameter

The volume of cellular patterns (V_{c0}) was calculated by subtracting the cavity volume from the total EB volume:

$V_{c0} = V_t - V_{cv} = \frac{\pi}{6}(d_t^3 - d_{cv}^3)$

To calculate the volume of the ExEn pattern (V_{ex}), we measured the ExEn height (h) at several points to obtain an average value, and then subtracted $2h$ from d_t to obtain V_1, where $V_1 = V_{cv} + V_{ep}$. The ExEn pattern volume was calculated as the difference between two volumes:

$$V_{ex} = V_t - V_1 = \frac{\pi}{6} d_t^3 - \frac{\pi}{6}(d_t - h)^3 = \frac{\pi}{6}(d_t^3 - (d_t - 2h)^3)$$

The volume of the Epi-l pattern (V_{ep}) was calculated as a difference between following volumes:

$$V_{ep} = V_{c0} - V_{ex} = \frac{\pi}{6}(d_t^3 - d_{cv}^3) - \frac{\pi}{6}(d_t^3 - (d_t - 2h)^3) = \frac{\pi}{6}((d_t - 2h)^3 - d_{cv}^3)$$

or $V_{ep} = V_1 - V_{cv} = \frac{\pi}{6}(d_t - h)^3 - \frac{\pi}{6} d_{cv}^3 = \frac{\pi}{6}((d_t - 2h)^3 - d_{cv}^3)$

Calculating normalized volume ratios for EB patterns

To analyze the EB growth rate and differentiation, sizes of EB patterns in each experimental series for each cell line were calculated by imaging and measuring of individual EBs (n=12 or n=24/ per group) at each studied stage (EB1, EB3, EB5, and EB10). After measuring the diameters of EB and the inner cavity and the height of ExEn layer in the images, corresponding volumes for individual EBs, the mean and SD values for the experimental groups, and then normalized values (relative to the volumes of EB1 or EB50) for each experimental series were calculated. To compare the experimental groups, we used the normalized mean values for three independent experiments using one-way ANOVA and Student-Newman-Keuls test for post hoc analysis. To analyze the volumetric ratios of EB10 to EB1, normalized data from three independent experiments were pooled and compared using two-tailed Student's unpaired t-test.

To calculate the volume ratios of actively proliferating domains, the mean values of the percentages of EdU-labeled cells and volume values of cell domains ($V_{c0} = V_t - V_{cv}$) for individual EBs at each stage for each cell line were used for each experimental series (Fig. 2I,2J). Data were normalized relative to ESB1 values and the mean values of normalized volumes/percentages of the EdU-labeled cell domain from three independent experiments were compared between cell lines at certain stages using one-way ANOVA and Student-Newman-Keuls post hoc test. To analyze the volumetric ratios of the cavity, EdU-labeled and unlabeled cell domains in EB5 and EB10, we compared the averaged normalized mean values of volumes for three experimental series using normalized stacked bar charts (Fig. 2K).

To study the volumetric ratios of three EB patterns (ExEn, Epi-l, and cavity), we calculated and compared the normalized mean values of volumes for each of the three patterns, which were calculated for individual EBs in groups in three independent experimental series as described above. Data were visualized and analyzed using normalized stacked bar charts (Fig. 6B and 7F).

Teratoma and teratocarcinoma assays

To study the in vivo differentiation potentials of EBs at day1 and day 10, the teratoma and teratocarcinoma assays were performed using immunodeficient nude mice. Nude mice at the age of 3-4 months were obtained from the Animal Breeding Facility-Branch "Pushchino" of the Institute of Bioorganic Chemistry, Russian Academy of Sciences (Moscow-Pushchino, Russia). Animal

maintenance and experiments were approved by the Ethics Committee of Institute of Developmental Biology of Russian Academy of Sciences and performed following the Russian Federation legislation (Order of the Ministry of Health and Social Development of the Russian Federation No 708n, August 28, 2010) based on the European Convention for the Protection of Vertebrate Animals Used for Experimental and Other Scientific Purposes.

For experiments, ten EBs at day 1 and day 10 of each cell line were transplanted under the kidney capsule of recipient mice (n=3). Before the transplantation procedures, the animals were anesthetized by an intraperitoneal injection of 50 mg/kg pentobarbitone sodium (Boehringer Ingelheim, Germany) and 10 mg/kg xylazine (Rometar, Spofa, Czech Republic). All surgery procedures were performed as previously described (Gordeeva, Nikonova, 2013). After 4-10 weeks, the animals were euthanized by cervical dislocation, and the developed tumors were removed.

For histological analysis, tumor samples were fixed in Bouin solution, dehydrated according to the standard histological method, and embedded into paraffin for sectioning. Histological sections were stained with hematoxylin and eosin, examined, and imaged under Leica DMRXA2 microscope with CD camera (Leica Microsystems, Germany).

Reference

Gordeeva O.F., Nikonova T.M. Development of Experimental Tumors Formed by Mouse and Human Embryonic Stem and Teratocarcinoma Cells after Subcutaneous and Intraperitoneal Transplantations into Immunodeficient and Immunocompetent Mice. Cell Transplant. 2013; 22(10):1901-14. doi: 10.3727/096368912X657837

Preparation of fluorescent protein tracers and live-cell analysis of their diffusion into EBs

To examine the free diffusion of proteins from culture media into the EBs, the fluorescent protein tracers were prepared via covalent labeling reaction of proteins with the fluorescein isothiocyanate isomer I (FITC, F7250, Sigma-Aldrich) dye and used for live-cell analysis. FITC-labeled protein tracers were prepared using the modified protocol for protein labeling (Modesti M.,

2018): soybean trypsin inhibitor (MW 20 kDa, T6522, Sigma-Aldrich) and ovalbumin (MW 42.7 kDa; A7641, Sigma-Aldrich) were dissolved in 50 mM phosphate buffer, pH 8.5 at a concentration of 10 mg/ml and centrifuged at 16000g for 30 min, the supernatants were cooled in an ice bath. FITC solution in dimethyl sulfoxide (10 mg/ml) was added to protein solutions at threefold molar excess of dye over the protein (3:1 MW). The reaction mixes were incubated at 4ºC overnight and then separated from the free dye by gel filtration using the columns with Sephadex G-10 (Pharmacia/GE Healthcare Life Sciences), which were equilibrated with a buffer containing 50 mM phosphate buffer pH 8.5, 100 mM NaCl, and 30% glycerol (all from Sigma-Aldrich), in accordance with the manufacturer's protocol. After protein concentration calculating, prepared solutions of fluorescently labeled proteins were diluted at the final concentration of 2mg/ml, aliquoted, and stored at -20°C.

For live-cell analysis, the fluorescent protein tracers were added to culture media without phenol red dye at a final concentration 2 μg/ml. The diffusion of the fluorescent protein tracers in the EBs was studied by confocal microscopy using an argon laser (488 nm) at 20% power and HCX PL APO CS 20.0x0.70 IMM objective.

Reference.

Modesti M. (2018) Fluorescent Labeling of Proteins. In: Peterman E. (eds) Single Molecule Analysis. Methods in Molecular Biology, vol 1665, p. 115-134. Humana Press, New York, NY. https://doi.org/10.1007/978-1-4939-7271-5_6

Supplementary Tables

Supplementary Table 1. Real-time reverse transcription polymerase chain reaction (qRT-PCR) primers.

Gene	Accession no.	Primer sequences	Amplicon (bp)
Hprt	NM_013556.2	5' ttgggcttacctcactgctttc 3'<br>5' ctaatcacgacgctgggactg 3'	125
Oct4	NM_013633.2	5' caccctgggcgttctctttg 3'<br>5' gttctcattgttgtcggcttcc 3'	142
Nanog	NM_028016	5' aactctcctccattctgaacctga 3'<br>5' ggtgctgagcccttctgaatc 3'	136
Mvh	NM_010029.2<br>NM_001145885.1	5' aggaatgccatcaaaggaacaac 3'<br>5' gcccaacagcgacaaacaag 3'	119
Gata4	NM_008092	5' tctcactatgggcacagcag 3'<br>5' gggacagcttcagagcagac 3'	100
Pax6	NM_013627.6	5' taccagtgtctaccagccaatcc 3'<br>5' gcacgagtatgaggaggtctga 3'	193
Bry	NM_009309.2	5' atgctgcctgtgagtcataac 3'<br>5' cgtgtgcgtcagtggtgtg 3'	177
ActivinA	NM_002192	5'tggagcagacctcggagatcatcac3'<br>5'ttggtcctggttctgttagccttgg3'	160
Nodal	NM_013611	5'gcgagtgtcctaaccctgtg3'<br>5'atgctcagtggcttggtc3'	136
Lefty1	NM_010094	5'tgtgtgctctttgcttcctctg3'<br>5'gcagtgaacaatatgaaggacagag3'	123
Tgfb1	NM_011577	5'caattcctggcgttaccttgg3'<br>5'ccctgtattccgtctccttgg3'	120
Bmp4	NM_007554	5'tctggtctccgtccctgatg3'<br>5'cgctccgaatggcactacg3'	175
Gdf3	NM_008108	5'gatgagtgtgggtgtgggtag3'<br>5'gtccgattcaagagagcataagc3'	109

Supplementary Table 2. EB shape descriptors for circularity/roundness analysis.

EB1	View	Area	StdDev	Major	Minor	Angel	Circularity	Skew	AR	Roundness	Solidity
N1 total	V1	19000.39	19.09	157.98	153.13	156.66	0.93	0.74	1.03	0.97	0.99
	V2	19101.73	19.67	158.63	153.32	173.56	0.90	0.76	1.04	0.97	0.99
	V3	19026.88	19.53	159.97	151.44	33.98	0.94	0.97	1.06	0.95	0.99
mean		**19043.00**	**19.43**	**158.86**	**152.63**	**121.40**	**0.92**	**0.82**	**1.04**	**0.96**	**0.99**
N2 total	V1	15135.91	17.54	144.99	132.92	135.70	0.94	0.48	1.09	0.92	0.99
	V2	15067.98	17.57	145.09	132.23	143.43	0.92	0.54	1.10	0.91	0.99
	V3	15083.62	19.71	141.83	135.41	27.16	0.93	0.87	1.05	0.96	0.99
mean		**15095.84**	**18.27**	**143.97**	**133.52**	**102.10**	**0.93**	**0.63**	**1.08**	**0.93**	**0.99**
N3 total	V1	14698.015	16.317	138.27	135.349	89.029	0.944	0.996	1.02	0.979	0.987
	V2	14670.082	16.262	140.7	132.754	88.758	0.933	0.827	1.06	0.944	0.989
	V3	14809.822	18.91	140.69	134.028	1.468	0.798	0.828	1.05	0.953	0.982
mean		**14725.97**	**17.16**	**139.89**	**134.04**	**59.75**	**0.89**	**0.88**	**1.04**	**0.96**	**0.99**
N4 total	V1	17833.643	21.623	152.41	148.986	105.75	0.926	0.779	1.02	0.978	0.983
	V2	17714.871	20.97	153.28	147.149	132.27	0.882	0.678	1.04	0.96	0.984
	V3	17597.112	21.681	153.54	145.93	20.165	0.882	1.076	1.05	0.95	0.991
mean		**17715.21**	**21.42**	**153.07**	**147.36**	**86.06**	**0.90**	**0.84**	**1.04**	**0.96**	**0.99**
N5 total	V1	18961.521	19.356	157.83	152.967	9.304	0.907	0.973	1.03	0.969	0.986
	V2	18462.122	18.438	169.54	138.654	170.57	0.875	0.725	1.22	0.818	0.991
	V3	18590.191	20.418	171.07	138.367	174	0.81	0.85	1.24	0.809	0.992
mean		**18671.28**	**19.40**	**166.14**	**143.33**	**117.96**	**0.86**	**0.85**	**1.16**	**0.87**	**0.99**
**Mean EB1**		**17050.26**	**19.14**	**152.39**	**142.18**	**97.45**	**0.90**	**0.81**	**1.07**	**0.94**	**0.99**

Supplementary Table 2. EB shape descriptors for circularity/roundness analysis (continued).

EB5	View	Area	StdDev	Major	Minor	Angel	Circularity	Skew	AR	Roundness	Solidity
N1 total	V1	49806.77	45.23	259.61	244.27	86.39	0.90	-0.04	1.06	0.94	0.99
	V2	49153.00	39.94	251.67	248.67	115.50	0.87	-0.50	1.01	0.99	0.99
	V3	49243.49	45.09	255.25	245.63	95.64	0.96	0.02	1.04	0.96	0.99
mean		**49401.09**	**43.42**	**255.51**	**246.19**	**99.18**	**0.91**	**-0.17**	**1.04**	**0.96**	**0.99**
N2 total	V1	59080.10	45.82	279.17	269.45	113.08	0.87	-0.20	1.04	0.97	0.99
	V2	57995.87	41.66	283.56	260.42	8.42	0.95	-0.20	1.09	0.92	0.99
	V3	58462.24	40.67	282.92	263.10	160.30	0.91	-0.27	1.08	0.93	0.99
mean		**58512.74**	**42.72**	**281.88**	**264.32**	**93.93**	**0.91**	**-0.22**	**1.07**	**0.94**	**0.99**
N3 total	V1	49710.83	40.74	257.92	245.41	110.51	0.90	-0.21	1.05	0.95	0.99
	V2	48630.18	40.92	254.58	243.21	156.59	0.95	-0.01	1.05	0.96	0.99
	V3	50117.68	41.01	261.04	244.45	73.61	0.83	0.28	1.07	0.94	0.99
mean		**49486.23**	**40.89**	**257.85**	**244.36**	**113.57**	**0.89**	**0.02**	**1.06**	**0.95**	**0.99**
N4 total	V1	67576.53	45.29	295.82	290.86	44.54	0.95	-0.21	1.02	0.98	0.99
	V2	68365.21	46.33	307.73	282.87	116.60	0.95	0.33	1.09	0.92	0.99
	V3	67251.22	44.65	301.12	284.37	98.45	0.72	0.29	1.06	0.94	0.98
mean		**67730.99**	**45.42**	**301.55**	**286.03**	**86.53**	**0.87**	**0.14**	**1.05**	**0.95**	**0.99**
N5 total	V1	47796.26	40.46	251.66	241.82	179.29	0.86	0.00	1.04	0.96	0.99
	V2	50473.72	43.01	263.48	243.91	34.18	0.93	0.36	1.08	0.93	0.99
	V3	46040.06	39.48	251.89	232.72	147.19	0.89	0.03	1.08	0.92	0.99
mean		**48103.35**	**40.99**	**255.68**	**239.48**	**120.22**	**0.89**	**0.13**	**1.07**	**0.94**	**0.99**
**Mean EB5 total**		**54646.88**	**42.69**	**270.49**	**256.08**	**102.69**	**0.90**	**-0.02**	**1.06**	**0.95**	**0.99**
N1 cavity	V1	12854.63	16.53	132.09	123.91	44.17	0.89	0.53	1.07	0.94	0.99
	V2	12615.57	18.08	131.21	122.42	55.28	0.82	0.84	1.07	0.93	0.99
	V3	12116.83	8.66	129.18	119.43	169.98	0.88	0.46	1.08	0.93	0.99
mean		**12529.01**	**14.42**	**130.82**	**121.92**	**89.81**	**0.86**	**0.61**	**1.07**	**0.93**	**0.99**
N2 cavity	V1	16264.84	16.30	145.83	142.01	41.21	0.94	1.11	1.03	0.97	0.99
	V2	15460.28	12.83	145.18	135.59	173.08	0.89	1.00	1.07	0.93	0.99
	V3	15527.00	13.26	145.23	136.13	154.21	0.92	1.06	1.07	0.94	0.99
mean		**15750.71**	**14.13**	**145.41**	**137.91**	**122.84**	**0.92**	**1.05**	**1.06**	**0.95**	**0.99**
N3 cavity	V1	12397.56	15.29	132.21	119.40	122.89	0.84	0.42	1.11	0.90	0.98
	V2	10532.31	15.49	123.02	109.01	52.63	0.89	1.05	1.13	0.89	0.99
	V3	13076.34	11.83	136.08	122.35	68.70	0.95	0.95	1.11	0.90	0.99
mean		**12002.07**	**14.20**	**130.43**	**116.92**	**81.41**	**0.89**	**0.81**	**1.12**	**0.90**	**0.98**
N4 cavity	V1	18047.69	15.31	155.25	148.02	147.01	0.93	0.75	1.05	0.95	0.99
	V2	16234.96	9.49	158.40	130.50	144.07	0.87	0.78	1.21	0.82	0.98
	V3	17482.00	16.51	153.39	145.11	78.12	0.92	1.12	1.06	0.95	0.98
mean		**17254.88**	**13.77**	**155.68**	**141.21**	**123.07**	**0.91**	**0.88**	**1.11**	**0.91**	**0.98**
N5 cavity	V1	10636.88	16.58	127.26	106.42	42.74	0.92	1.25	1.20	0.84	0.99
	V2	12005.77	24.10	135.80	112.56	25.09	0.77	1.34	1.21	0.83	0.98
	V3	12614.29	19.91	130.65	122.93	42.94	0.94	0.86	1.06	0.94	0.98
mean		**11752.31**	**20.20**	**131.24**	**113.97**	**36.92**	**0.88**	**1.15**	**1.16**	**0.87**	**0.98**
**Mean EB5 cavity**		**13857.80**	**15.34**	**138.72**	**126.39**	**90.81**	**0.89**	**0.90**	**1.10**	**0.91**	**0.99**

Supplementary Table 2. EB shape descriptors for circularity/roundness analysis (continued).

EB10	View	Area	StdDev	Major	Minor	Angel	Circularity	Skew	AR	Roundnes	Solidity
N1 total	V1	608795.13	51.46	955.65	811.11	175.82	0.87	0.35	1.18	0.85	0.97
	V2	612083.78	48.37	897.19	868.63	8.26	0.82	0.10	1.03	0.97	0.96
	V3	594297.36	47.70	905.72	835.45	39.51	0.92	0.12	1.08	0.92	0.98
mean		**605058.75**	**49.17**	**919.52**	**838.40**	**74.53**	**0.87**	**0.19**	**1.10**	**0.91**	**0.97**
N2 total	V1	571651.09	50.76	856.67	849.63	125.19	0.97	0.18	1.01	0.99	1.00
	V2	596232.75	44.89	919.25	825.84	172.30	0.92	0.23	1.11	0.90	0.98
	V3	596184.97	53.17	881.65	860.98	99.46	0.95	0.17	1.02	0.98	1.00
mean		**588022.94**	**49.61**	**885.86**	**845.48**	**132.32**	**0.95**	**0.19**	**1.05**	**0.96**	**0.99**
N3 total	V1	629571.03	68.69	955.00	839.37	154.41	0.93	0.32	1.14	0.88	1.00
	V2	604926.47	53.07	920.28	836.94	35.83	0.90	0.54	1.10	0.91	0.99
	V3	608506.32	53.29	899.35	861.48	24.73	0.88	0.53	1.04	0.96	0.98
mean		**614334.61**	**58.35**	**924.88**	**845.93**	**71.66**	**0.91**	**0.46**	**1.09**	**0.92**	**0.99**
N4 total	V1	572752.18	35.40	917.39	794.92	152.09	0.95	0.59	1.15	0.87	0.99
	V2	568475.79	35.55	874.37	827.80	175.29	0.88	0.71	1.06	0.95	0.97
	V3	583716.29	35.43	882.89	841.80	170.97	0.90	0.65	1.05	0.95	0.98
mean		**574981.42**	**35.46**	**891.55**	**821.50**	**166.12**	**0.91**	**0.65**	**1.09**	**0.92**	**0.98**
N5 total	V1	622472.13	32.55	913.88	867.24	157.40	0.93	0.39	1.05	0.95	0.99
	V2	623978.82	29.37	927.98	856.14	14.47	0.96	0.89	1.08	0.92	0.99
	V3	643483.49	24.44	946.02	866.06	157.01	0.94	0.92	1.09	0.92	0.99
mean		**629978.14**	**28.78**	**929.29**	**863.14**	**109.62**	**0.94**	**0.73**	**1.08**	**0.93**	**0.99**
**Mean EB10 total**		**602475.17**	**44.27**	**910.22**	**842.89**	**110.85**	**0.91**	**0.44**	**1.08**	**0.93**	**0.98**
N1 cavity	V1	237203.76	20.28	600.82	502.67	166.80	0.76	1.12	1.20	0.84	0.99
	V2	217784.03	19.19	535.82	517.51	122.54	0.94	0.77	1.04	0.97	0.99
	V3	225678.49	22.48	551.96	520.58	50.50	0.94	0.93	1.06	0.94	0.99
mean		**226888.76**	**20.65**	**562.87**	**513.59**	**113.28**	**0.88**	**0.94**	**1.10**	**0.92**	**0.99**
N2 cavity	V1	214842.85	25.40	540.72	505.89	76.85	0.75	0.77	1.07	0.94	0.99
	V2	228894.40	24.80	579.95	502.53	171.31	0.95	0.51	1.15	0.87	1.00
	V3	227097.31	26.10	580.78	497.86	99.14	0.95	0.66	1.17	0.86	0.99
mean		**223611.52**	**25.43**	**567.15**	**502.09**	**115.77**	**0.88**	**0.65**	**1.13**	**0.89**	**0.99**
N3 cavity	V1	265613.45	21.95	632.81	534.43	160.53	0.95	1.25	1.18	0.85	0.99
	V2	242963.47	23.36	589.01	525.20	142.22	0.93	0.98	1.12	0.89	0.99
	V3	264828.25	26.15	598.73	563.18	15.06	0.93	0.97	1.06	0.94	0.99
mean		**257801.73**	**23.82**	**606.85**	**540.94**	**105.94**	**0.94**	**1.07**	**1.12**	**0.89**	**0.99**
N4 cavity	V1	241490.5	21.382	574.91	534.821	2.695	0.97	0.612	1.08	0.93	0.994
	V2	229929.92	21.568	569.43	514.121	154.27	0.934	0.38	1.11	0.903	0.996
	V3	237198.72	20.897	556.13	543.055	13.703	0.977	0.238	1.02	0.976	0.994
mean		**236206.38**	**21.28**	**566.83**	**530.67**	**56.89**	**0.96**	**0.41**	**1.07**	**0.94**	**0.99**
N5 cavity	V1	241622.43	21.327	579.79	530.609	138.45	0.859	0.21	1.09	0.915	0.995
	V2	233429.07	10.843	573.13	518.576	177.45	0.933	0.516	1.11	0.905	0.995
	V3	252011.84	18.158	593.8	540.368	132.87	0.88	0.316	1.1	0.91	0.997
mean		**242354.45**	**16.78**	**582.24**	**529.85**	**149.59**	**0.89**	**0.35**	**1.10**	**0.91**	**1.00**
**Mean EB10 cavity**		**237372.57**	**21.59**	**577.19**	**523.43**	**108.29**	**0.91**	**0.68**	**1.10**	**0.91**	**0.99**
EB1-EB5 total							**0.687**		**0.576**	**0.521**	**1.0**
EB1-EB10 total							**0.415**		**0.698**	**0.692**	**0.172**
EB5-EB10 total							**0.203**		**0.0497 ***	**0.0656**	**0.172**
EB5-EB10 cavity							**0.391**		**1.0**	**0.907**	**0.0353 ***

Supplementary Figures

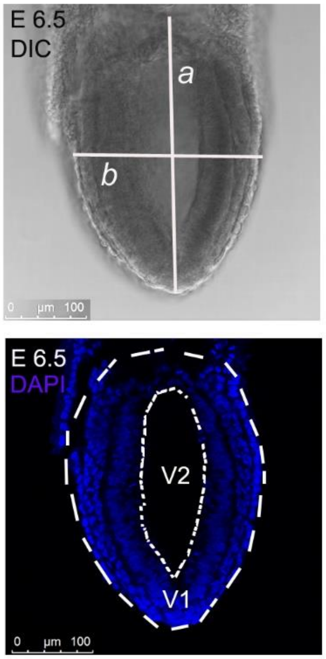

Embryonic Stage	Total volume of Epiblast, ExEn, and Pro-amniotic cavity (V1), µm3	Pro-amniotic cavity volume (V2), µm3	V2 / V1 ratio, %
E 6.0 n=3	26012.39	4398.23	17
	36155.54	6953.39	19
	34587.36	8600.63	25
Mean ± SD	32251.76	6650.75	**20 ± 0.03**
E 6.5 n=3	37648.85	10178.76	27
	37898.08	12007.17	32
	36868.68	8372.34	23
Mean ± SD	37471.87	10186.09	**27 ± 0.03**

Supplementary Figure 1. Volumetric ratios of the pro-amniotic cavity to total embryoblast at E6.0-6.5 stages. Representative confocal images of embryoblast with visceral ExEn of the E6.5 embryos. Nuclei were stained with Hoechst 33342 (Hst). Two diameters of the spheroid (a and b) on the middle plane of confocal z-stacks using differential interference contrast (DIC) and the 405 laser with HCX PL APO CS 20.0x0.70 IMM UV objective. The table shows the volumes of total embryoblasts with ExEn (V1) and pro-amniotic cavities (V2) of the corresponding embryos (n = 3) at stages E6.0 and E6.5 and their percentages (V2/V1), as well as means ± s.d. The volumes of the embryoblasts and pro-amniotic cavities were calculated as the volume of the spheroid (rotational ellipsoid) after measuring the diameters in two directions (a and b) using the formula: $V = 4\pi ab^2/3$.

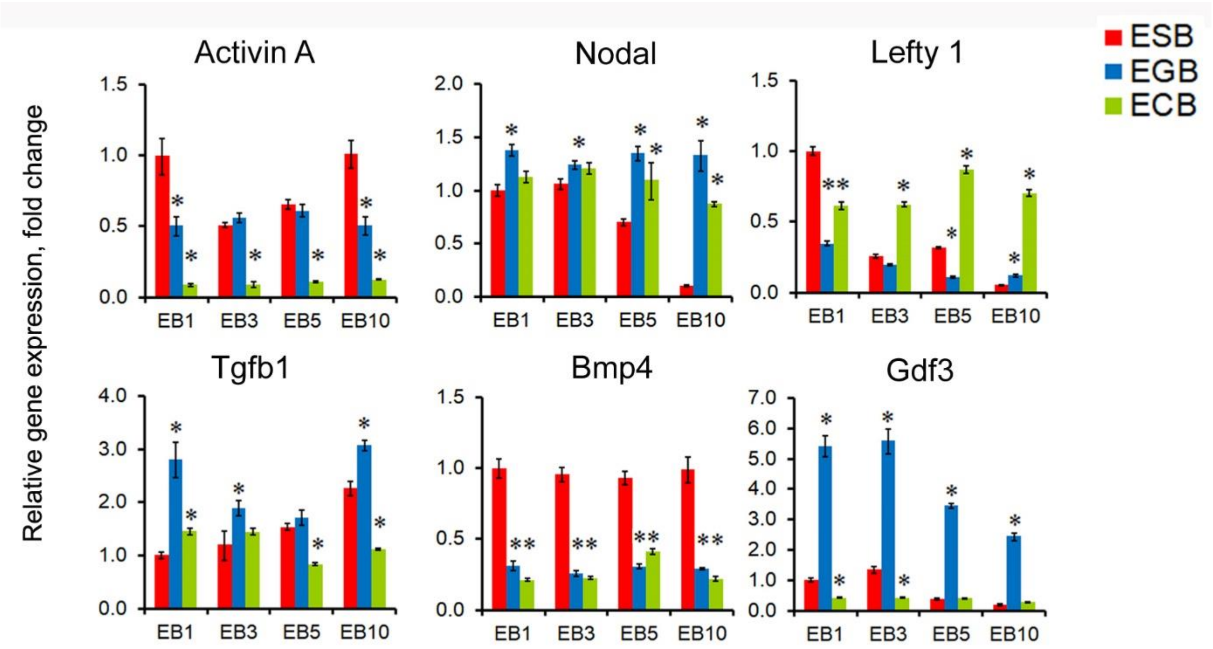

Supplementary Figure 2. Expression profiles of TGFβ family factors in differentiating ESBs, EGBs, and ECBs. Relative gene expression for each factor was evaluated relative to expression levels in ESB1. Data are shown as means ± s.d. from three experiments. * *P<0.05*, ANOVA. The most significant differences in expression levels were found for most factors (Activin, Lefty1, Tgfβ1, Bmp4 and Gdf3) between ESBs, EGBs and ECBs.

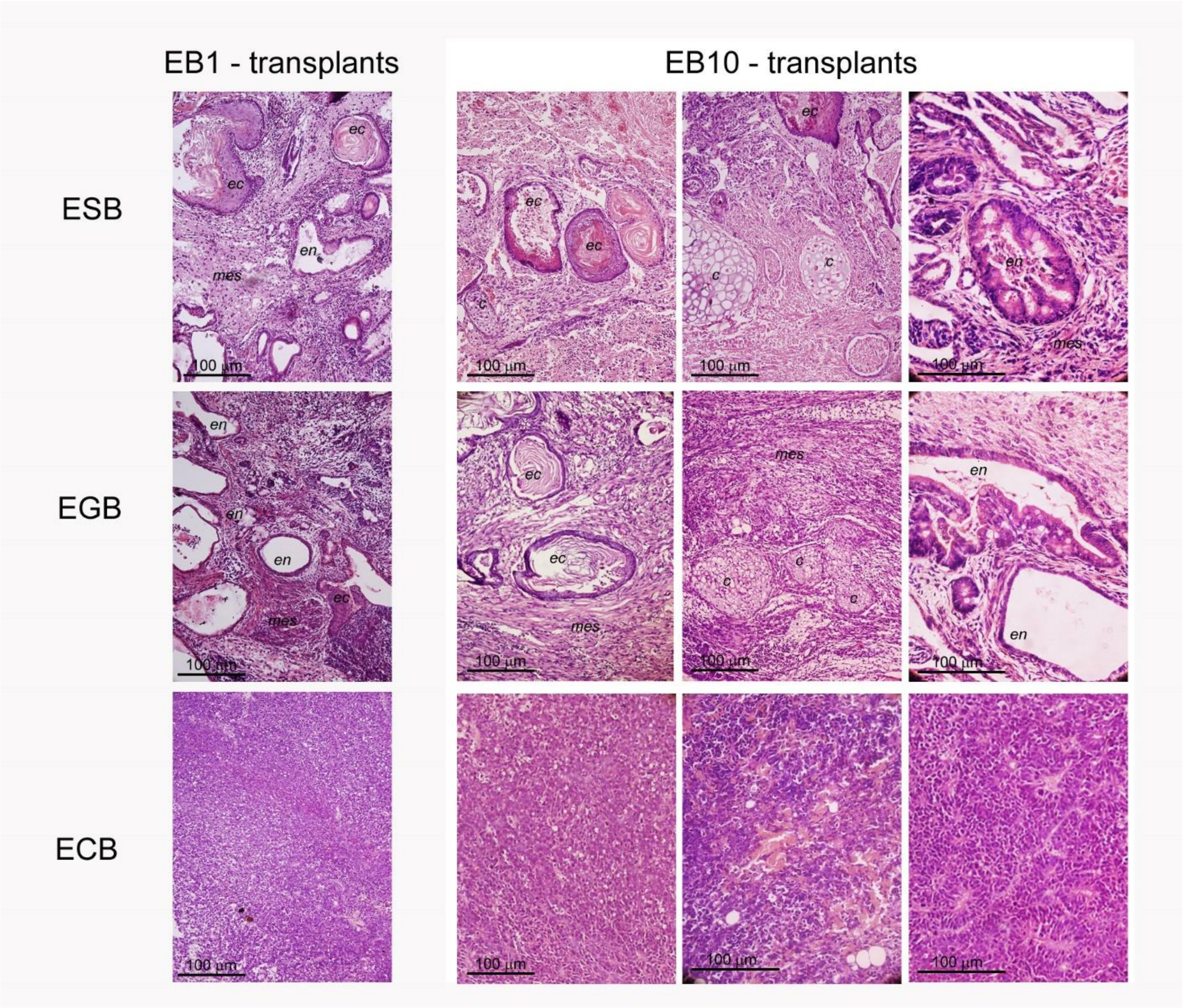

Supplementary Figure 3. Teratomas and teratocarcinomas developed after ESB, EGB, and ECB transplantations. Histological sections of teratomas and teratocarcinomas developed after transplantations of ESB, EGB and ECB at EB1 and EB10 stages. Differentiation patterns of tumors formed after transplantation of the EB1 and EB10 into nude mice were strictly consistent with those of undifferentiated cells of parental lines: ESBs and EGBs formed teratomas with the derivatives of the three germ layers and ECBs formed undifferentiated teratocarcinoma tumors. In teratomas formed by EB1 and EB10, the ectodermal sloughing and keratinized epithelium (ec), endodermal secreting epithelium (en) and mesodermal derivatives (cartilage (c), muscle and fibroblasts (mes)) were found. No significant differences were observed in the composition of cell derivatives for tumors generated by EB1 and EB10 for each cell line.

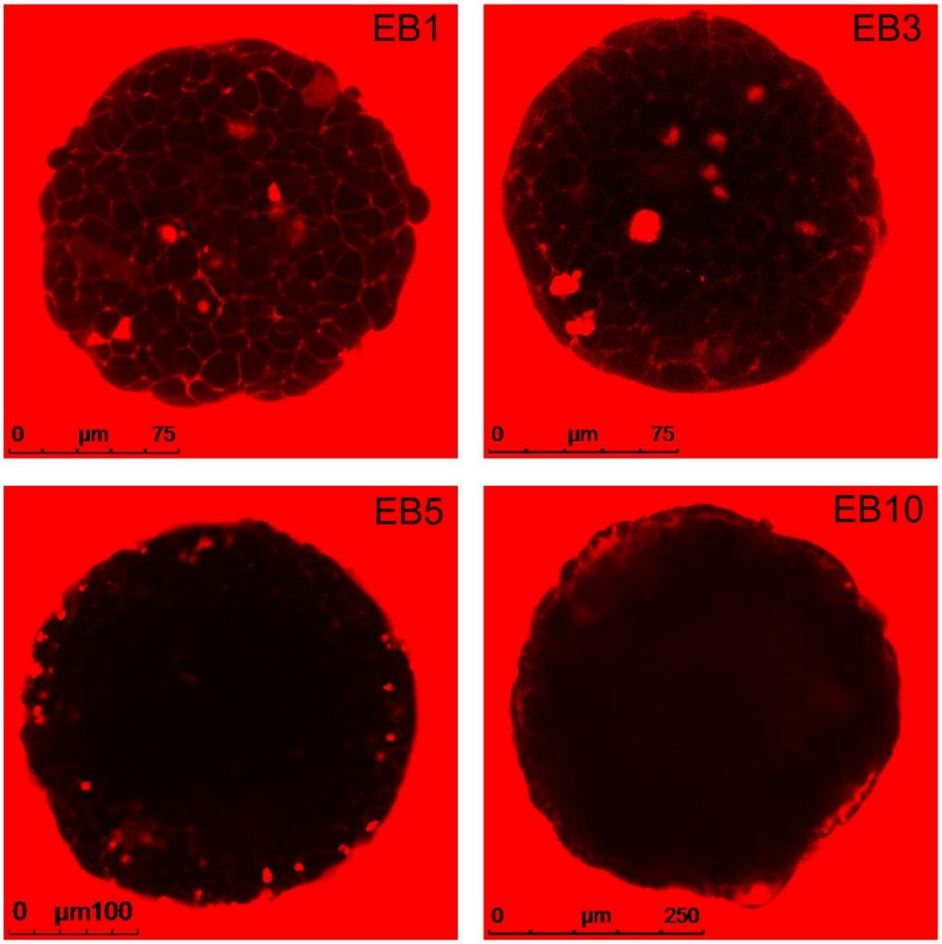

Supplementary Figure 4. RITC diffusion analysis in differentiating ESBs. Representative images of live cell analysis for the RITC diffusion in ESB 1-10. Free diffusion of the RITC after 15 min exposure at the EB1 and EB3 stages and 60 min at the EB5 and EB10 stages was analyzed using a Leica TSC SP5 confocal microscope with a temperature control system. Free diffusion was detected in the intercellular space of between all cells of EB1 and EB3, and dead cells were stained by RITC. Partial diffusion in the intercellular space of between outer cells and dead cell staining was detected in ESB5 after RITC exposure. No RITC diffusion was detected in the inner layers of ESB10 except partial diffusion in the outer ExEn cell layer.

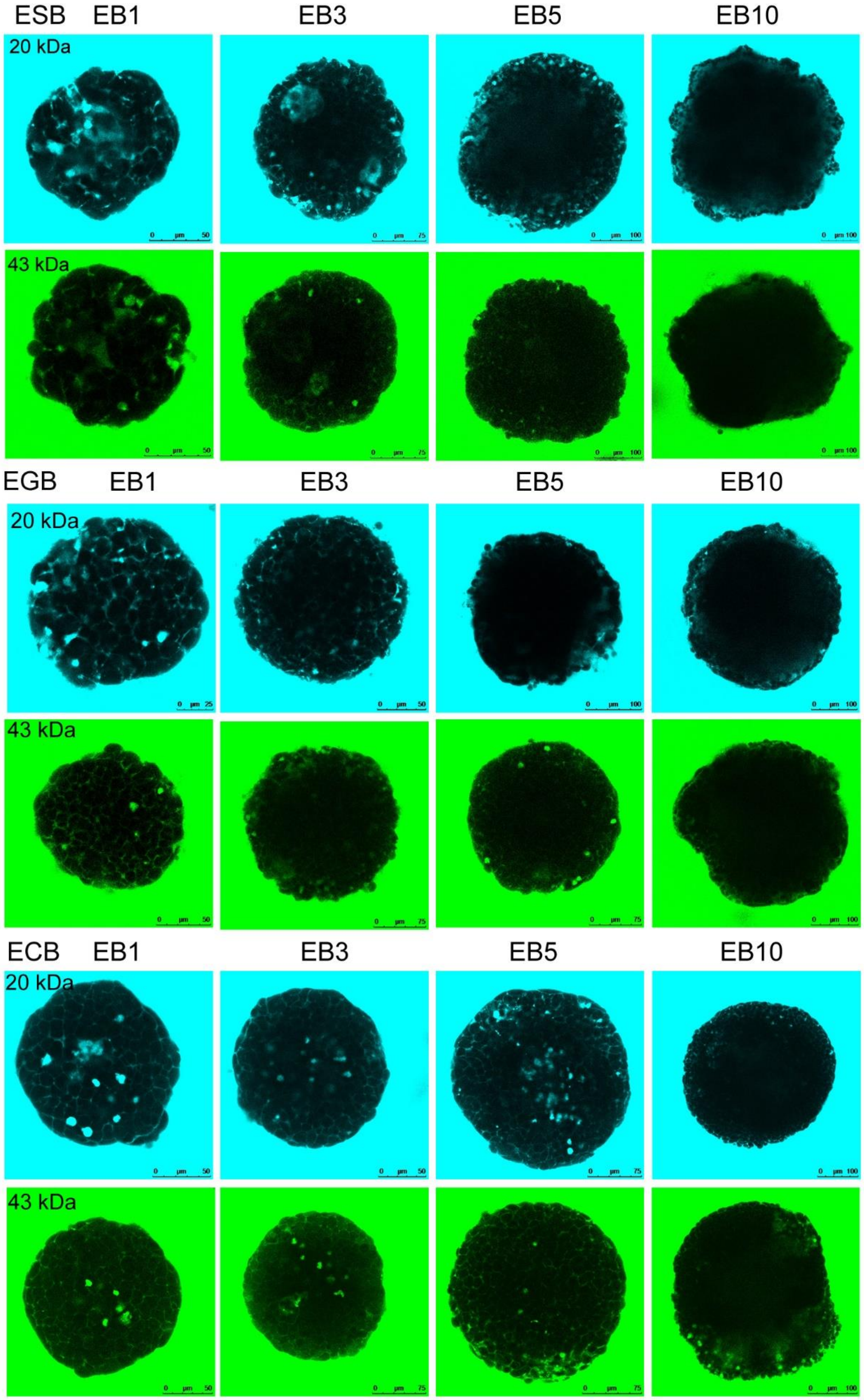
ESB
EB1
EB3
EB5
EB10
20 kDa
43 kDa
EGB
EB1
EB3
EB5
EB10
20 kDa
43 kDa
ECB
EB1
EB3
EB5
EB10
20 kDa
43 kDa

Supplementary Figure 5. Protein diffusion analysis in differentiating ESBs, EGBs, and ECBs. Representative images of live-cell analysis for the protein tracer diffusion in ESB, EGBs, ECBs at the EB1- EB10 stages. Free diffusion of the protein tracers with molecular weights 20 and 43 kDa conjugated with FITC fluorescence dye (see Suppl. Methods) was studied for ESBs, EGBs, and ECBs within 15, 30, and 60 min using a Leica TSC SP5 confocal microscope with a temperature control system (at 37ºC). Free diffusion was detected in the intercellular space of EB1 and EB3, partial diffusion in EB5 and no free diffusion was detected in inner layers of EB10 (except local leaks).

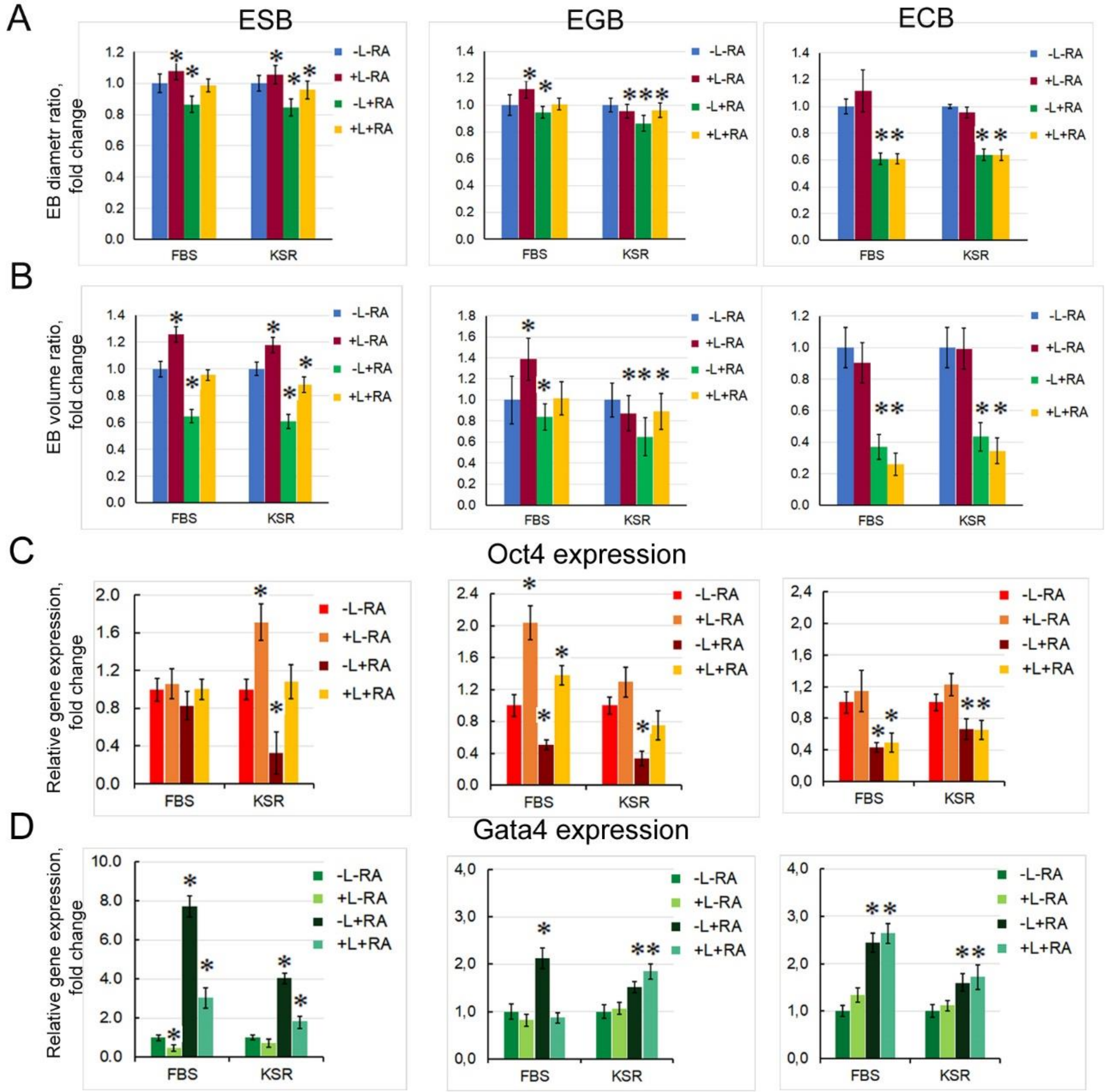

Supplementary Figure 6. Growth rate and expression of Oct4 and Gata4 in the ESBs, EGBs, and ECBs cultured in FBS- and KSR-media and treated with retinoic acid (RA) and leukemia growth factor (LIF). (A, B) Diameter and volume ratios for ESBs, EGBs, and ECBs at day 5 cultured in FBS- and KSR-media treated with different combinations of RA (10^{-6} M) and LIF (1000 U/ml). A significant decrease in diameters and volumes was found for all EBs after exposure to RA, while LIF increased the growth of ESBs and EGBs, but not EBCs. RA and LIF combination treatment led to the annihilation of their single growth effects. The data are shown as means±s.d. from two experiments. * *P<0.05*, ANOVA. (C) Expression of Oct4 and Gata4 in differentiating ESBs, EGBs, and ECBs grown in KSR- and FBS-media and treated with RA and LIF. Relative gene expression for each gene was evaluated relative to expression levels in untreated EB-FBS or EB-KSR, relatively, for each cell line. The data are presented as means±s.d. from three experiments. * *P<0.05*, ANOVA.

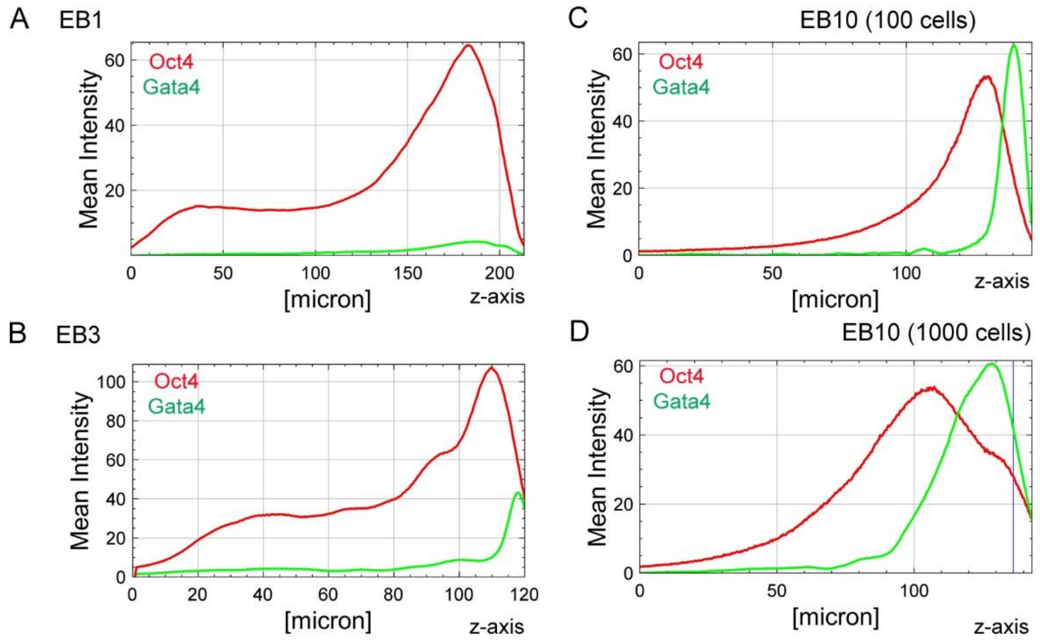

Supplementary Figure 7. Spatial trends for the Oct4 and Gata4 expression intensity along the z-axis scanning of EB1, EB3, and EB10. (A, B) Representative plots of spatial trends of the intensity (a.u) of the expression of Oct4 and Gata4 along the z-axis scanning in EB1 and EB3 stacks demonstrate changes in Gata4 expression in the surface cells. The x-axis shows the z-axis of the scan from the EB center to the surface. (C,D) Representative plots of spatial trends of the Oct4 and Gata4 expression intensity (a.u) in EB100 and EB1000 indicate the similar distributions of expression of these marker proteins along the z-axis scanning. Distributions of Gata4 and Oct4 staining intensity indicate ExEn and Epi-l patterns of different sizes in EB100 and EB1000. The regional immunofluorescence intensity of Oct4 and Gata4 expression was determined along the z-axis using the ROI/Multi plot tool of ImageJ/Fiji software. The line plots of z-axis profiles of Oct4 and Gata4 expression were generated with the Plot z-axis profile tool to analyze the intensity values through the z-axis within an ROI (100x100 μm) on the stacks for EBs at different stages.

Video files' descriptions

Movie 1. 3D reconstructed models of the uniform spatial pattern of the Oct4 expressing cells in ESB at day 1 (EB1). Confocal z-stack images were processed using the 3D Viewer tool of ImageJ/Fiji software. Oct4 - red (Alexa 594).

Movie 2. Emerging ExEn cells in E3.5 blastocyst. 3D reconstructed models of spatial patterns of the pluripotent (Oct4) and ExEn (Gata4) cells in mouse embryos at the E3.5 stage. Confocal z-stack images were processed using the 3D Viewer tool of ImageJ/Fiji software. Oct4 - red (Alexa 594); Gata4 - green (Alexa 488).

Movie 3. 3D reconstructed models of spatial patterns of the Epi-l (Oct4) and ExEn (Gata4) cells in ESB at day 3 (EB3). The first ExEn cells on the EB surface appear stochastically. The self-organizing ExEn pattern in EB3 is similar to that in E3.5 blastocyst. Confocal z-stack images were processed using the 3D Viewer tool of ImageJ/Fiji software. Oct4 - red (Alexa 594); Gata4 - green (Alexa 488).

Movie 4. Self-organizing ExEn pattern in E5.0 blastocyst. 3D reconstructed models of spatial patterns of the pluripotent (Oct4) and ExEn (Gata4) cells in mouse embryos at the E4.5 stage. Confocal z-stack images were processed using the 3D Viewer tool of ImageJ/Fiji software. Oct4 - red (Alexa 594); Gata4 - green (Alexa 488).

Movie 5. 3D reconstructed models of spatial patterns of the Epi-l (Oct4) and ExEn (Gata4) cells in ESB at day 3 (EB3). The ExEn layer is compacted and gradually covers the surface of the EB. Confocal z-stack images were processed using the 3D Viewer tool of ImageJ/Fiji software. Oct4 - red (Alexa 594); Gata4 - green (Alexa 488).

Movie 6. 3D reconstructed models of spatiotemporal patterns of the Epi-l (Oct4) and ExEn (Gata4) cells in ESB at day 5 (EB5). The ExEn pattern is fully formed since the ExEn cell layer covers the entire surface of the EB. Confocal z-stack images were processed using the 3D Viewer tool of ImageJ/Fiji software. Oct4 - red (Alexa 594); Gata4 - green (Alexa 488).

Movie 7. 3D reconstructed models of spatiotemporal patterns of the Epi-l (Oct4) and ExEn (Gata4) cells in ESB at day 10 (EB10). Asymmetric overgrowths of the ExEn on the EB10 surface. Confocal z-stack images were processed using the 3D Viewer tool of ImageJ/Fiji software. Oct4 - red (Alexa 594); Gata4 - green (Alexa 488).
